# The bifunctional enzyme, GenB4, catalyzes the last step of gentamicin 3′,4′-di-deoxygenation via reduction and transamination activities

**DOI:** 10.1186/s12934-020-01317-0

**Published:** 2020-03-10

**Authors:** Xiaotang Chen, Hui Zhang, Shaotong Zhou, Mingjun Bi, Shizhou Qi, Huiyuan Gao, Xianpu Ni, Huanzhang Xia

**Affiliations:** 1 School of Life Science and Biopharmaceutics, Shenyang Pharmaceutical University, No.103 Wenhua Road, Shenyang, Liaoning China; 2 School of Traditional Chinese Medicine, Shenyang Pharmaceutical University, No.103 Wenhua Road, Shenyang, Liaoning China

**Keywords:** Gentamicin, Di-deoxygenation, GenB4, Reduction activity, Transamination activity

## Abstract

**Background:**

New semi-synthetic aminoglycoside antibiotics generally use chemical modifications to avoid inactivity from pathogens. One of the most used modifications is 3′,4′-di-deoxygenation, which imitates the structure of gentamicin. However, the mechanism of di-deoxygenation has not been clearly elucidated.

**Results:**

Here, we report that the bifunctional enzyme, GenB4, catalyzes the last step of gentamicin 3′,4′-di-deoxygenation via reduction and transamination activities. Following disruption of *gen*B4 in wild-type *M. echinospora*, its products accumulated in 6′-deamino-6′-oxoverdamicin (**1**), verdamicin C2a (**2**), and its epimer, verdamicin C2 (**3**). Following disruption of *gen*B4 in *M. echinospora* Δ*gen*K, its products accumulated in sisomicin (**4**) and 6′-*N*-methylsisomicin (**5**, G-52). Following in vitro catalytic reactions, GenB4 transformed sisomicin (**4**) to gentamicin C1a (**9**) and transformed verdamicin C2a (**2**) and its epimer, verdamicin C2 (**3**), to gentamicin C2a (**11**) and gentamicin C2 (**12**), respectively.

**Conclusion:**

This finding indicated that in addition to its transamination activity, GenB4 exhibits specific 4′,5′ double-bond reducing activity and is responsible for the last step of gentamicin 3′,4′-di-deoxygenation. Taken together, we propose three new intermediates that may refine and supplement the specific biosynthetic pathway of gentamicin C components and lay the foundation for the complete elucidation of di-deoxygenation mechanisms.

## Background

Drug-resistant pathogens have spread dramatically across the world and have become a major threat to public health [1]. Studies have found that several aminoglycosides can resist multiple drug-resistant pathogens, especially for Gram-negative bacteria [2, 3]. One example is gentamicin, which contains 3′,4′-di-deoxygenation structures and avoids modification of *o*-phosphotransferases (APHs) and *o*-adenyltransferases (ANTs) to 3′,4′-hydroxy groups [4]. Since the hydroxyl group of the aminoglycoside antibiotic is the main attack site of resistant-bacteria inactivation enzymes, these hydroxyl-deoxygenated atoms cannot be chemically modified. New semi-synthetic antibiotics used in clinical applications also use this modification. For example, arbekacin and dibekacin contain 3′,4′-di-deoxygenation structures obtained by chemical syntheses. Therefore, elucidation of the mechanisms of gentamicin 3′,4′-di-deoxygenation has theoretical and practical value for the development of novel aminoglycoside antibiotics.

**Fig. 1:**
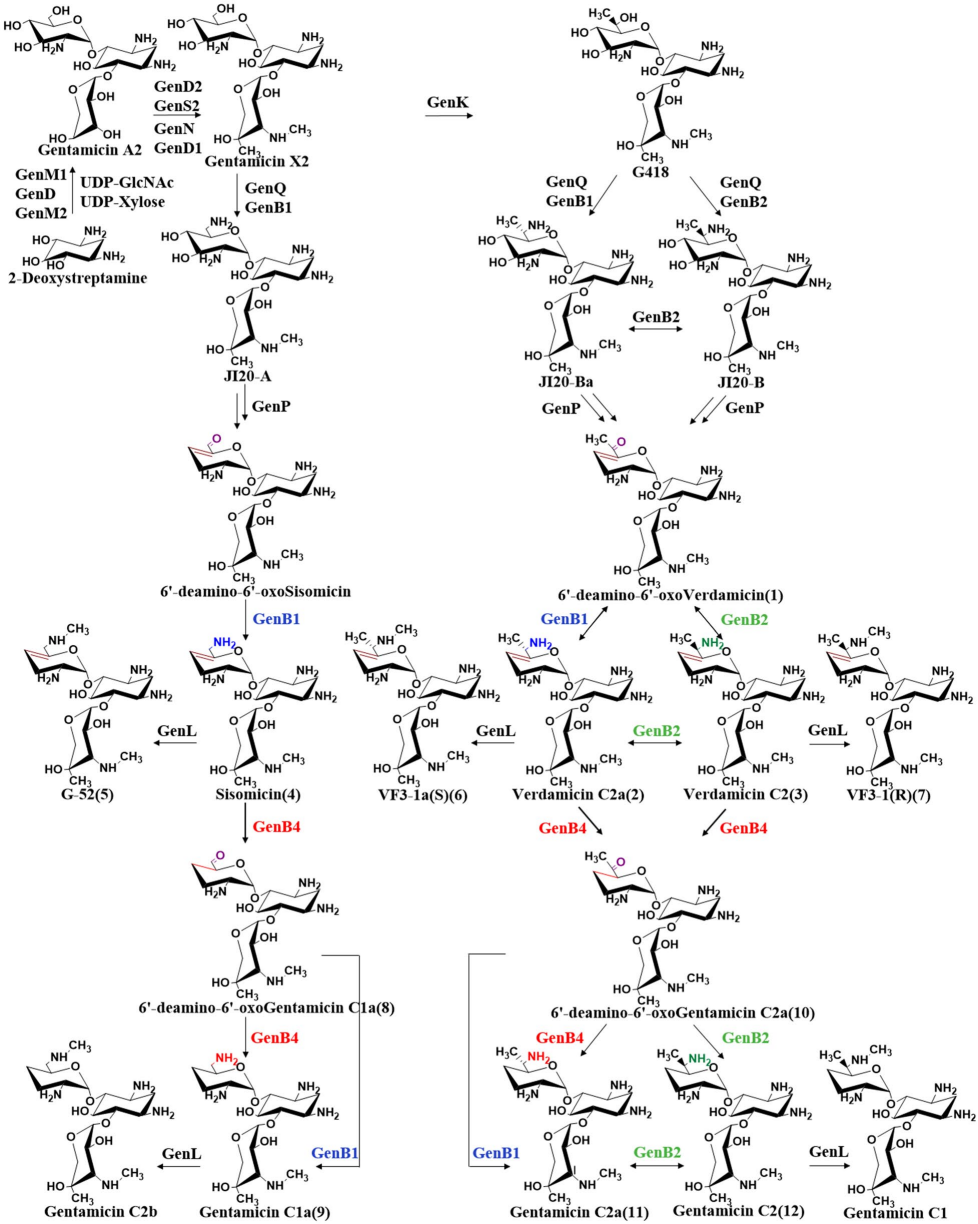
Biosynthetic pathway of gentamicin

Gentamicin is a complex mixture of four major compounds: gentamicin C1a, C2, C2a, and C1 [5]. Gentamicin belongs to the group of 4,6-disubstituted 2-deoxystreptamine (DOS) aminoglycoside antibiotics. The biosynthetic pathway of gentamicin has been mostly elucidated, and it is known that gentamicin A2 is the first pseudotrisaccharide intermediate. Via a series of enzyme-catalyzed reactions, gentamicin A2 is transformed to gentamicin X2, which is the key branch of the next set of parallel routes [6]. The C6′ methyltransferase, GenK, catalyzes gentamicin X2 to G418 [7]. GenQ and GenB1/GenB2 catalyze gentamicin X2 and G418 via dehydrogenation and transamination separately to produce JI-20A, JI-20Ba, and JI-20B. JI-20A, JI-20Ba, and JI-20B are then catalyzed by a series of dideoxygenases to form gentamicin C1a, gentamicin C2a, and gentamicin C2, respectively. Gentamicin C1a and gentamicin C2 are further catalyzed by the N6′ methyltransferase, GenL, to form gentamicin C2b and gentamicin C1 [8]. The biosynthetic pathway of gentamicin is shown in Fig. 1. Importantly, the mechanism of di-deoxygenation has still not been fully elucidated. Lei et al. demonstrated that the phosphotransferase, GenP, catalyzes the first step of 3′,4′-di-deoxygenation in gentamicin biosynthesis [9]. Guo et al. disrupted *gen*B3 and *gen*B4 separately in wild-type *M. echinospora* and the products accumulated into JI-20A and JI-20B; additionally, they demonstrated that GenB1, GenB2, GenB3, and GenB4 are pyridoxal-5′-phosphate (PLP)-dependent aminotransferases [6].

## Results and discussion

### Inactivation of *gen*B4 in *M. echinospora*

**Fig. 2:**
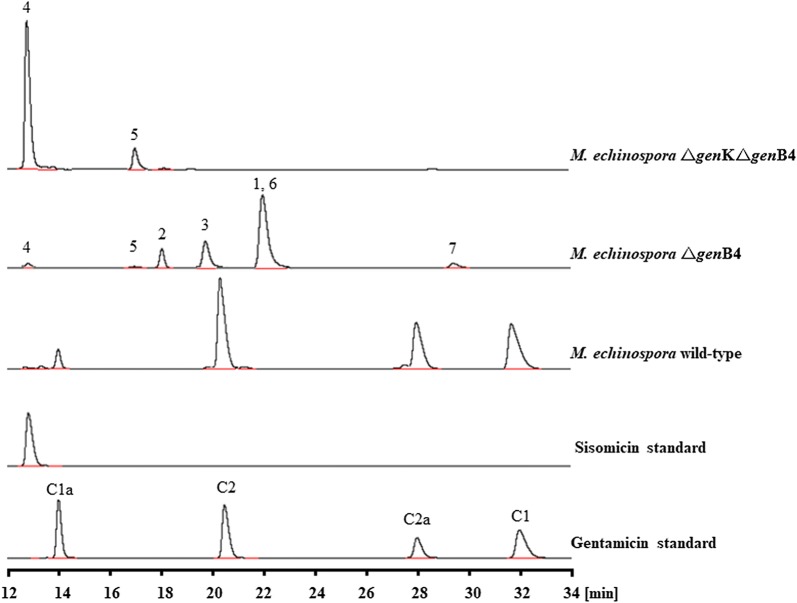
HPLC-ELSD analysis of fermentation production by *M. echinospora* wild type and mutants

To investigate the role of *gen*B4, a 939-bp internal DNA fragment was deleted in-frame with the pD2925B4 plasmid in wild-type *M. echinospora* and *M. echinospora* Δ*gen*K (Additional file 1: Fig. S1a). The mutants were confirmed by PCR (Additional file 1: Fig. S1b and c). The disrupting strains were fermented, and their products were analyzed through high-performance liquid chromatography with evaporative light-scattering detection (HPLC-ELSD). Compared to that in the wild-type strain, *M. echinospora* Δ*gen*B4 did not produce any of the gentamicin C complex. Rather, its products accumulated as intermediates such as (**1**), (**2**), and (**3**) as well as minor components in the form of (**6**) and (**7**) (Fig. 2). Additionally, (**1)** and (**6)** had the same retention time in HPLC-ELSD during separation by cation-exchange chromatography (Additional file 2: Fig. S2). The disrupting strain *M. echinospora* Δ*gen*KΔ*gen*B4 accumulated in (**4**) and (**5**) (Fig. 2). To determine the structures of these new products, the intermediates were separated, purified, and analyzed through mass and nuclear magnetic resonance (NMR) spectroscopic analyses. The exact mass of intermediate (**4**) was 448.2770 ([M+H]^+^) (Additional file 3: Fig. S3a), and its retention time was consistent with that of sisomicin. The exact mass of intermediate (**5**) was 462.2944 ([M+H]^+^) (Additional file 3: Fig. S3b), which was consistent with that of 6′-*N*-methylsisomicin (**5**, G-52) [10–12]. Hence, we hypothesized that in the other parallel pathway of gentamicin biosynthesis, the *gen*B4 disrupting strain would accumulate as verdamicin and 6′-*N*-methylverdamicin (VF3-1) [13–16]. Interestingly, the exact mass of intermediate (**2**) was 462.3000 ([M+H]^+^) (Additional file 3: Fig. S3c), and the exact mass of (**3**) was 462.3005 ([M+H]^+^) (Additional file 3: Fig. S3d), both of which were consistent with that of verdamicin. The exact mass of intermediate (**6**) was 476.3194 ([M+H]^+^) (Additional file 4: Fig. S4a), and the exact mass of (**7**) was 476.3145 ([M+H]^+^) (Additional file 4: Fig. S4b), both of which were consistent with that of VF3-1. Hanessian et al. reported a synthesis of verdamicin C2a and its congener, C2, from sisomicin in vitro in 2008, at which time this synthesis was first mentioned and its chiral isomerization structure was named [17]. Our present in vitro catalytic findings demonstrate that (**2**) was verdamicin C2a and (**3**) was verdamicin C2. Based on the retention times of HPLC-ELSD, we hypothesized that (**6**) was VF3-1a(S) and (**7**) was VF3-1(R). The exact mass of intermediate (**1**) was 461.2698 ([M+H]^+^) (Additional file 4: Fig. S4c) and could be reduced by NaBH_4_ (Additional file 2: Fig. S2); ^1^H NMR and ^13^C NMR spectroscopic analyses identified that intermediate (**1**) was 6′-deamino-6′-oxoverdamicin (Additional file 5: Fig. S5).

### Complementation of the *gen*B4 disrupting strain

To further determine the in vivo role of *gen*B4, we constructed the *gen*B4 complement plasmid, pEAP1B4, which contained the entire *gen*B4 under the control of the P*hrd*B promoter (Additional file 6: Fig. S6a). After sequence verification, pEAP1B4 was introduced into Δ*gen*B4 and Δ*gen*KΔ*gen*B4 to construct the complementation strains, Δ*gen*B4::*gen*B4 and Δ*gen*KΔ*gen*B4::*gen*B4, respectively. The exconjugants were confirmed by PCR (Additional file 6: Fig. S6b). As shown in Additional file 7: Fig. S7, the complementation strain Δ*gen*B4::*gen*B4 restored production of the gentamicin C complex such that they were the same as those in the wild-type strain, and Δ*gen*KΔ*gen*B4::*gen*B4 restored the productions of gentamicin C1a (**9**) and C2b such that they were the same as those in the original strain of Δ*gen*K.

### GenB4 catalyzes sisomicin (4) to gentamicin C1a (9)

**Fig. 3:**
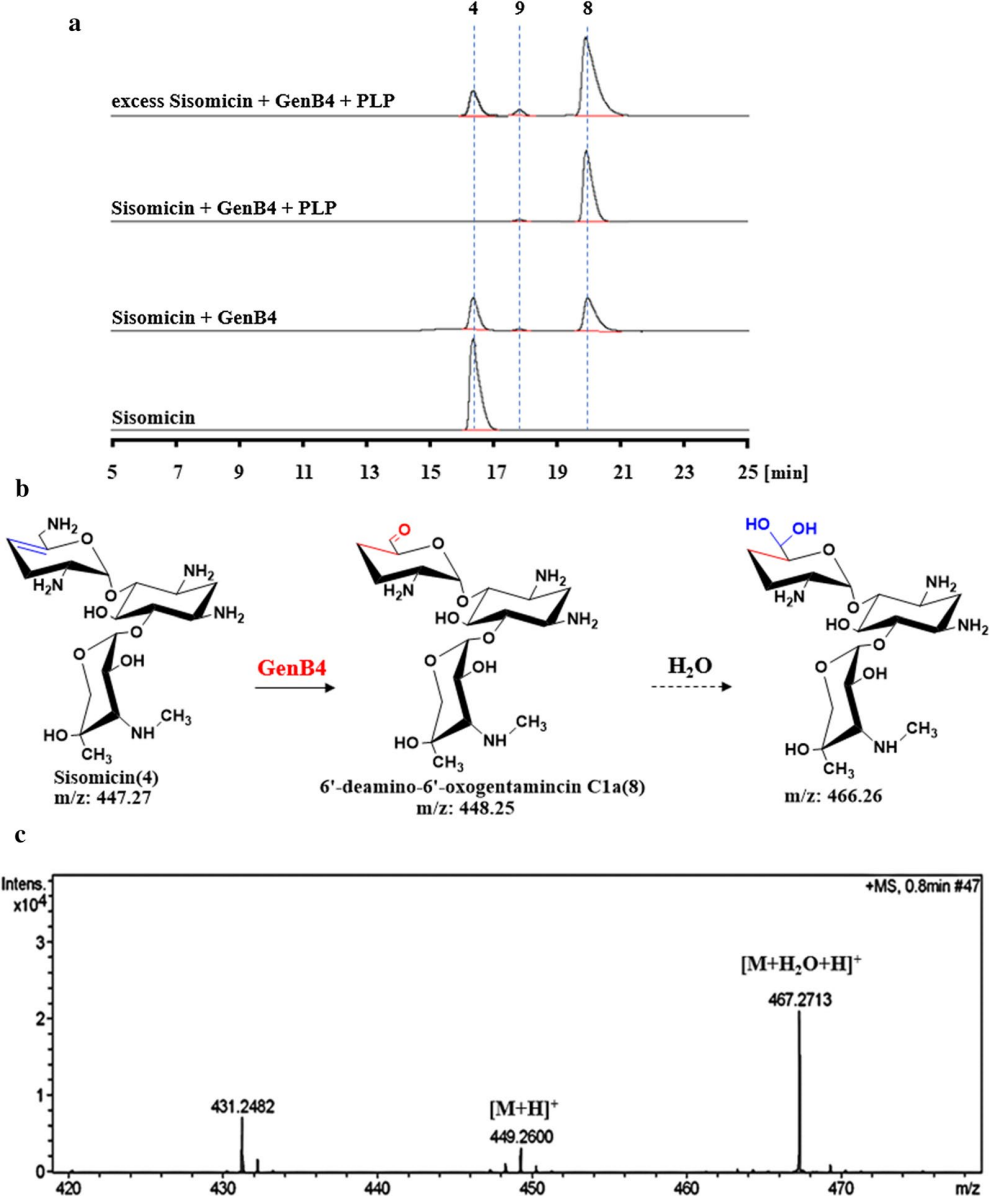
Analysis of GenB4-catalyzed reactions. **a** HPLC-ELSD analysis of GenB4 enzymatic assays. **b** Proposed structure of the catalysate. **c** Mass-spectra analysis of compound (**8**). [M+H]^+^*m*/*z* 449, [M+H_2_O+H]^+^*m*/*z* 467

**Fig. 4:**
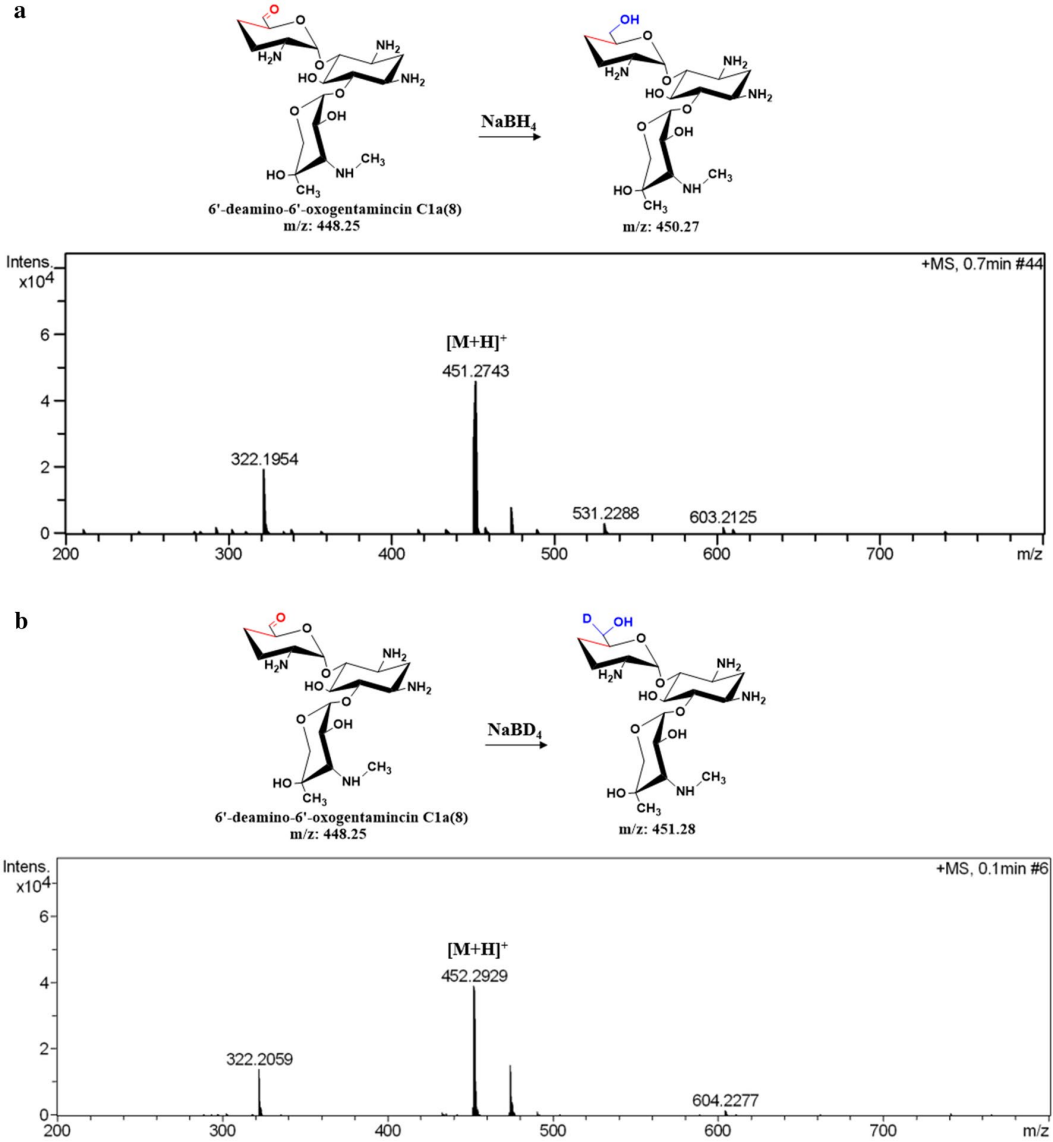
Mass-spectra analyses of compound (**8**) reduced with NaBH_4_ or NaBD_4_. **a** Mass-spectra analysis of compound (**8**) reduced with NaBH_4_. **b** Mass-spectra analysis of compound (**8**) reduced with NaBD_4_

For in vitro assays, soluble N-His6-tagged GenB4 proteins were produced in *E. coli* BL21(DE3) harboring the pET28a-based plasmid pET28aB4 (Table 1) and were purified using a Ni2^+^ affinity column (GE Healthcare) via standard protocols. The resulting recombinant proteins appeared light yellow in color and exhibited an absorbance spectrum characteristic of pyridoxal-5′-phosphate (PLP) binding (Additional file 8: Fig. S8). For in vitro assays, we used sisomicin (**4**) (purchased by Sigma-Aldrich) as a substrate and GenB4 to transform it into a new compound (**8**). Additionally, the rate was largely improved by supplementing exogenous PLP (Fig. 3a). However, compound (**8**) was not stable, as it readily underwent hydration in solution. According to its mass spectrum analysis, we predicted that it had an aldehyde structure (Fig. 3) [18, 19]. Then, compound (**8**) was treated with NaBH_4_ or NaBD_4_ to clarify the expected aldehyde formation [18]. Mass, ^1^H NMR, and ^13^C NMR spectroscopic analyses demonstrated that compound (**8**) was 6′-deamino-6′-oxogentamicin C1a (Fig. 4 and Additional file 9: Fig. S9).

**Table 1:** Strains and plasmids used in the present study

Strains or plasmids	Relevant characteristics	Reference or source
Strains		
*E. coli* TOP10	Host strain for cloning	Invitrogen
*E. coli* ET12567/pUZ8002	Methylation defective, strain used in *E. coli*-*streptomyces* intergeneric conjugation	[25]
*E. coli* BL21(DE3)	Host strain for protein expression	Novagen
*M. echinospora*	Wild-type strain, gentamicin C1a, C2, C2a, and C1 producer	This lab
*M. echinospora* Δ*gen*K	*M. echinospora* in which *gen*K was disrupted	This lab
*M. echinospora* Δ*gen*B4	*M. echinospora* in which *gen*B4 was disrupted	This study
*M. echinospora* Δ*gen*KΔ*gen*B4	*M. echinospora* in which *gen*K and *gen*B4 were disrupted	This study
*M. echinospora* Δ*gen*B4::Δ*gen*B4	Complementation of *gen*B4 in *M. echinospora* Δ*gen*B4	This study
*M. echinospora* Δ*gen*KΔ*gen*B4::Δ*gen*B4	Complementation of *gen*B4 in *M. echinospora* Δ*gen*KΔ*gen*B4	This study
Plasmids		
pIJ2925	Cloning vector for *E. coli ori*(pUC18), Amp^R^	[26]
pD2925	*E. coli*-*Streptomyces* shuttle vector, *oriT*(RP4), *ori*(pUC18), Amp^R^, Am^R^	[21]
pPT2925	pIJ2925 containing promoter *Phrd*B and terminator *To,* Amp^R^	This lab
pD2925B4	pD2925 containing upstream and downstream fragments of *gen*B4, Amp^R^, Am^R^	This study
pEAP1	*E. coli*-*Streptomyces* shuttle vector, *oriT*(RP4), *ori*(pUC18), *int*-*attP*(φC31), Amp^R^, Erm^R^	This lab
pEAP1B4	pEAP1 containing promoter *Phrd*B, *gen*B4 fragment and terminator *To*, Amp^R^, Erm^R^	This study
pET28a(+)	Protein expression vector used in *E. coli*, encoding N-terminal His-tag, Km^R^	Novagen
pET28aB4	pET28a(+) containing *gen*B4 fragment, Km^R^	This study
pET28aB1	pET28a(+) containing *gen*B1 fragment, Km^R^	This lab
pET28aB2	pET28a(+) containing *gen*B2 fragment, Km^R^	This lab
pET28aB3	pET28a(+) containing *gen*B3 fragment, Km^R^	This lab

Amp^R^ ampicillin resistance, Am^R^ ampramycin resistance, Erm^R^ erythromycin resistance, Km^R^ kanamycin resistance

Interestingly, the catalytic reaction produced a new minor component when we increased the amount of the substrate sisomicin (**4**) and supplemented exogenous PLP (Fig. 3a). As we expected, the new minor component was gentamicin C1a (**9**) (Additional file 10: Fig. S10). Then, different amino donors (L-Tyr, L-Lys, L-Gln, L-Met, and L-Glu) were added separately into the reaction systems to improve the transamination efficiency of 6′-deamino-6′-oxogentamicin C1a (**8**) to gentamicin C1a (**9**). The result showed that the yield of gentamicin C1a (**9**) increased significantly with L-Glu as an amino donor (Additional file 11: Fig. S11).

The process of the conversion of sisomicin (**4**) to gentamicin C1a (**9**) catalyzed by GenB4 was divided into two steps. In the first step, GenB4 catalyzed sisomicin (**4**) to 6′-deamino-6′-oxogentamicin C1a (**8**). In this reaction, the 4′,5′ double bond of sisomicin (**4**) was reduced, and the amino group at C6′ was transformed to an aldehyde group. The second step is the transamination of the 6′-deamino-6′-oxogentamicin C1a (**8**) to gentamicin C1a (**9**). We concluded that GenB4 was a bifunctional enzyme with reduction and transamination activities. Then, GenB1, GenB2, and GenB3 (i.e., three other characteristic aminotransferases of the gentamicin biosynthetic pathway) were also expressed and purified to examine the transamination of the 6′-deamino-6′-oxogentamicin C1a (**8**) to gentamicin C1a (**9**), among which GenB1 clearly showed the highest activity (Additional file 12: Fig. S12) [6, 20].

### GenB4 catalyzes verdamicin C2a (2) and verdamicin C2 (3) to gentamicin C2a (11) and gentamicin C2 (12), respectively

**Fig. 5:**
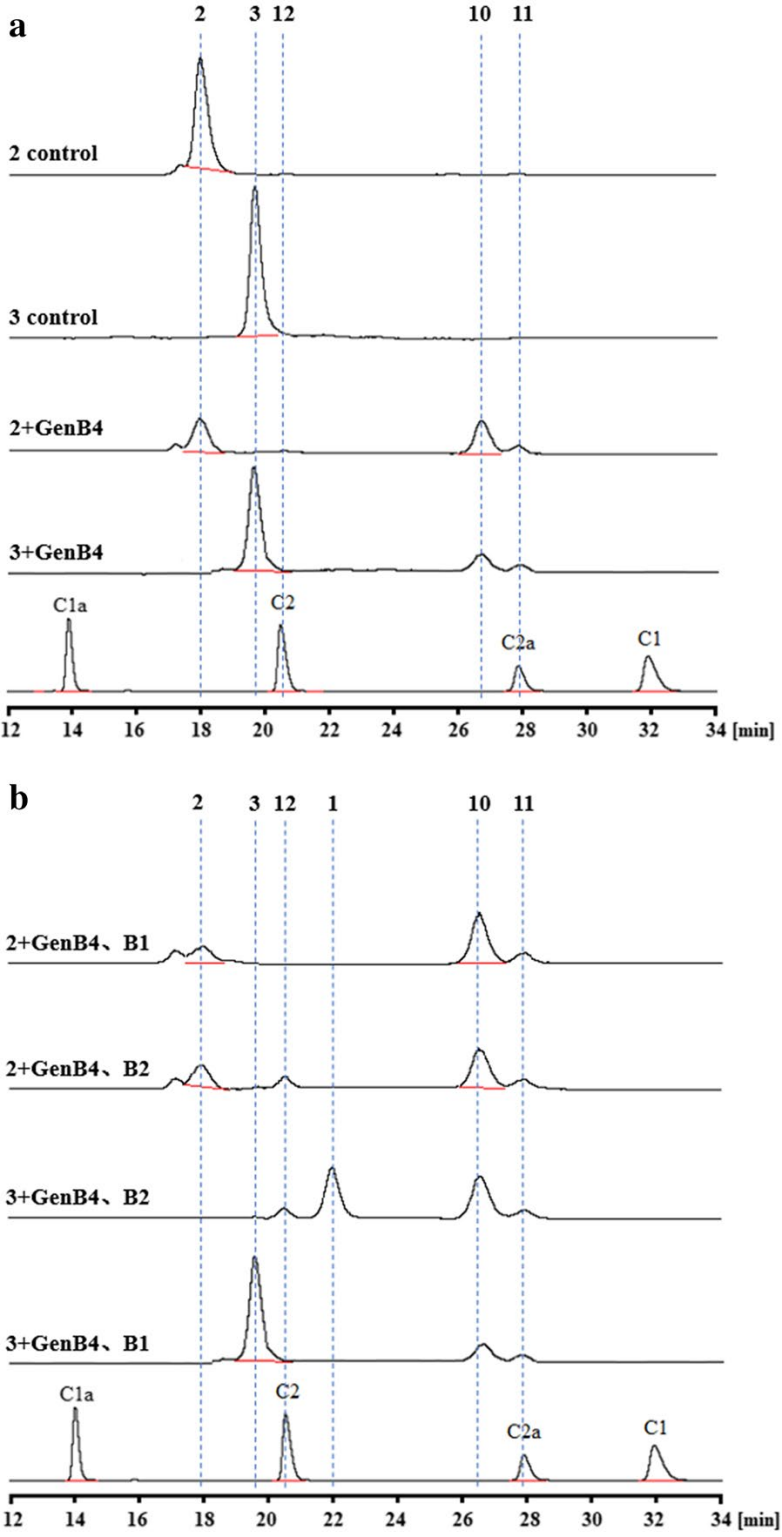
HPLC-ELSD analysis of GenB4-catalyzed verdamicin C2a (**2**) and verdamicin C2 (**3**) to gentamicin C2a (**11**) and gentamicin C2 (**12**)

In the other parallel pathway in our in vitro assays, (**2**) and (**3**) were separately used as substrates; GenB4 transformed them into new compounds (**10**) and (**11**), respectively (Fig. 5a). Additionally, (**11**) had the same retention time as that of gentamicin C2a in HPLC-ELSD, and its exact mass was 464.2827 ([M+H]^+^) (Additional file 13: Fig. S13a), which further confirmed that it was gentamicin C2a (**11**). The exact mass of (**10**) was 463.2822 ([M+H]^+^) (Additional file 13: Fig. S13b); upon referencing the catalytic process of sisomicin (**4**) to gentamicin C1a (**9**), we identified that (**10**) was 6′-deamino-6′-oxogentamicin C2a. Then, in the above reaction system, we separately added the aminotransferases, GenB1 and GenB2. The groups with GenB2 produced a new compound (**12**) that had the same retention time as that of gentamicin C2 (Fig. 5b). Importantly, when GenB4-GenB2 were combined to catalyze (**3**), the reaction efficiency was remarkably improved and produced the deamination product, 6′-deamino-6′-oxoverdamicin (**1**). Hence, we concluded that (**2**) was verdamicin C2a and that (**3**) was verdamicin C2 [21].

**Fig. 6:**
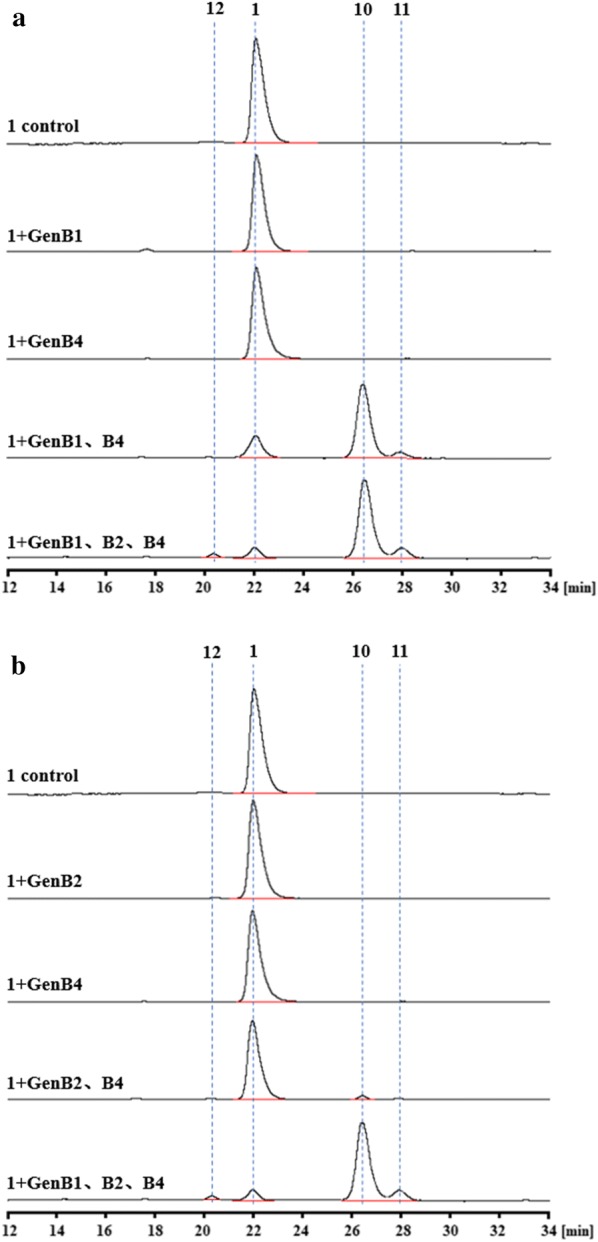
HPLC-ELSD analysis of 6′-deamino-6′-oxoverdamicin (**1**) catalyzed by GenB4

We also used 6′-deamino-6′-oxoverdamicin (**1**) as a substrate, but GenB4 was unable to transform it. Interestingly, when we used GenB1 or GenB2 to catalyze (**1**), no transamination reaction occurred in this situation either. It was only when either GenB1–GenB4 or GenB2–GenB4 was combined to catalyze (**1**) that products corresponding to the new compounds (**10**), (**11**), and (**12**) were yielded (Fig. 6). These findings indicate that in the biosynthetic pathway of gentamicin, (**1**) is upstream of (**2**) and (**3**). Additionally, only transamination of (**1**) to (**2**) and (**3**) can be recognized by GenB4. Therefore, we conclude that the 6′ amino group is an important recognition site for GenB4 reduction activity.

### Transformation between (**1**), (**2**), and (**3**) and a proposal of the new biosynthetic pathway

**Fig. 7:**
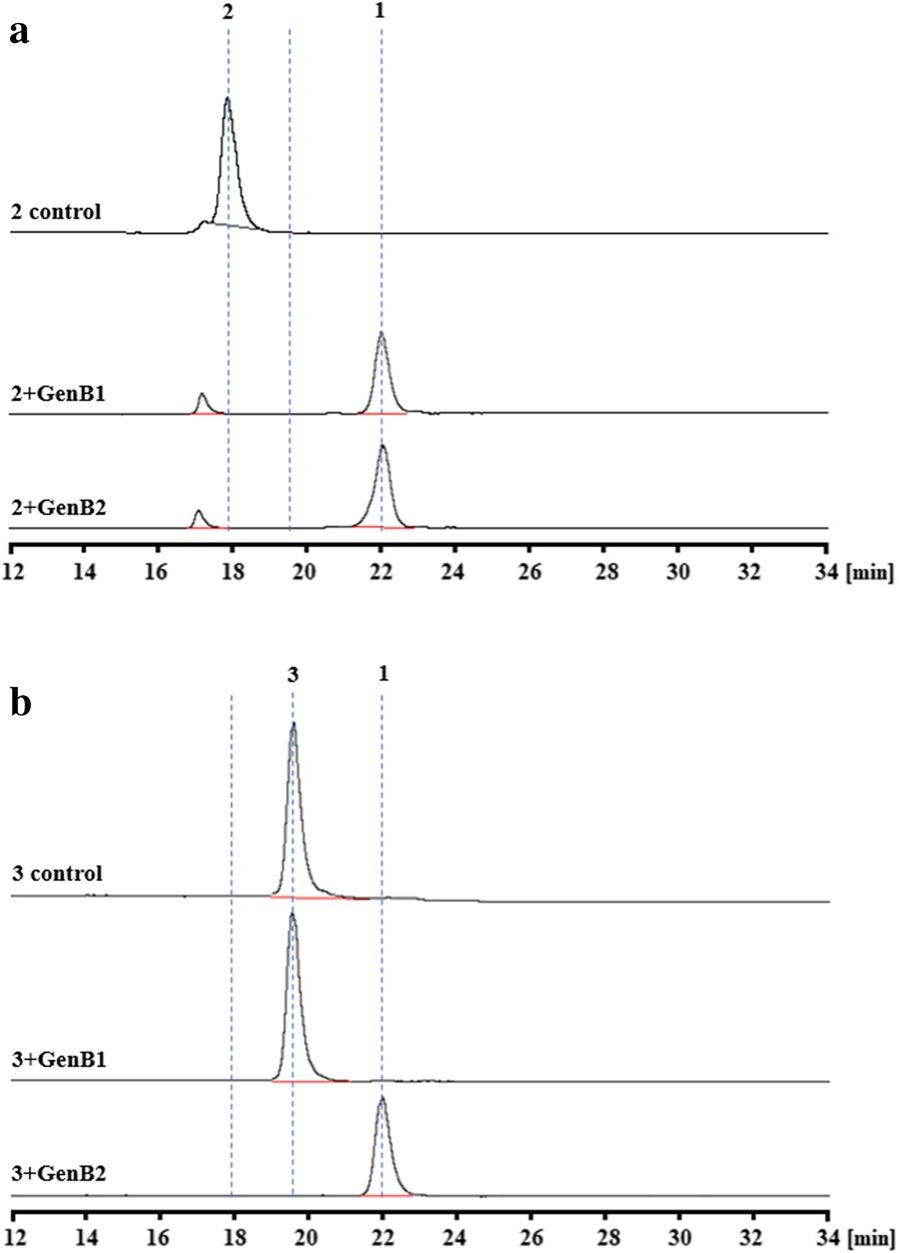
HPLC-ELSD analysis of the transformations of (**1**), (**2**), and (**3**)

The following experiments were completed to further elucidate the mutual conversion between (**1**), (**2**), and (**3**). Specifically, (**2**) was used as a substrate, GenB1 and GenB2 were added separately, and (**2**) was deaminated to generate (**1**). When (**3**) was used as a substrate, no reaction occurred when GenB1 was added. However, GenB2 efficiently transformed (**3**) to (**1**). Interestingly, no isomeric reactions occurred during the catalysis, and (**3**) was deaminated and transformed to (**1**) (Fig. 7). These findings indicated that in the balance of transamination and deamination reactions among (**1**), (**2**), and (**3**), deamination was more preferred. These findings also explain the accumulation of (**1**) in *M. echinospora* Δ*gen*B4.

In summary, our present findings have led us to propose a new supplement to the gentamicin 3′,4′-di-deoxygenation pathway. GenP and other enzymes catalyzed JI-20Ba and JI-20B to (**1**). Then, (**1**) was transaminated to (**2**) and its epimer, (**3**), via GenB1 and GenB2, respectively. Subsequently, (**2**) and (**3**) were reduced by GenB4 to produce the intermediate (**10**), which was further transaminated to (**11**) and its epimer, (**12**). Remarkably, only GenB2 catalyzed the transamination and deamination among (**1**), (**3**), and (**10**), (**12**). Both (**2**) and (**3**) can be methylated by GenL to generate (**6**) and its epimer (**7**). In the other parallel pathway, GenP and other enzymes catalyzed JI-20A to (**4**). Furthermore, we hypothesize that 6′-deamino-6′-oxosisomicin was not stable and that its lack of any steric hindrance of the 6′ methyl group made it easier to be transaminated to (**4**). Finally, (**4**) was reduced by GenB4 to produce (**8**), which was then further transaminated to (**9**). Collectively, the detailed biosynthetic pathway that we elucidated is shown in Fig. 1.

## Conclusions

In the present study, we disrupted *gen*B4 in wild-type *M. echinospora*, and its products accumulated as 6′-deamino-6′-oxoverdamicin (**1**), verdamicin C2a (**2**) and its epimer, verdamicin C2 (**3**). We also disrupted *gen*B4 in *M. echinospora* Δ*gen*K, and its products accumulated as sisomicin (**4**). Following in vitro catalytic reactions, GenB4 transformed sisomicin (**4**) to gentamicin C1a (**9**), and transformed verdamicin C2a (**2**) and its epimer, verdamicin C2 (**3**), to gentamicin C2a (**11**) and gentamicin C2 (**12**). Taken together, these findings demonstrate that GenB4 is a bifunctional enzyme with both reduction and transamination activities that are responsible for the last step of gentamicin 3′,4′-di-deoxygenation. Additionally, we provide the first proposal of the structures of intermediates (**1**), (**8**), and (**10**). Collectively, our present study refines and supplements the specific biosynthetic pathways of gentamicin C components, which lays the foundation for fully elucidating the mechanisms of di-deoxygenation. With clarifying genes and the unique biosynthetic pathway of gentamicin di-deoxygenation, deoxygenation enzyme complex will also be utilized for constructing strain producing semi-synthetic dibekacin at the same time. This work will lay the foundation for utilizing di-deoxygenation enzyme complex for biosynthesis semi-synthetic antibiotics like dibekacin or create new drugs with better bioactivity.

## Methods

### Bacterial strains, plasmids, and growth conditions

The strains and plasmids used in the present study are listed in Table 1. *Escherichia coli* DH5α was used as a cloning host and was grown on Luria–Bertani (LB) liquid or solid medium. Liquid ATCC172 was employed for *M. echinospora* vegetative growth [22]. The solid medium contained the following: soluble starch (10 g/L), MgSO_4_·7H_2_O (0.5 g/L), KNO_3_ (1 g/L), NaCl (0.5 g/L), asparagine (0.2 g/L), CaCO_3_ (1 g/L), wheat bran (10 g/L), K_2_HPO_4_·3H_2_O (0.3 g/L), and agar (15 g/L). The solid medium was used for *M. echinospora* sporulation, and conjugal transfer was performed on MS agar medium. The wild-type strain of *M. echinospora* and its derivative mutants were cultured by a two-stage fermentation at 34 °C using a seed medium that contained 10 g/L soluble starch, 15 g/L soya bean meal, 1 g/L glucose, 0.5 g/L KNO_3_, and 3 g/L CaCO_3_. After incubation at 34 °C for 36 h, 3 mL [10% (vol/vol)] of seed culture was used to incubate the fermentation medium, after which it was maintained at 34 °C with a shaking speed of 220 r/min for 5 days. The fermentation culture medium consisted of soluble starch (50 g/L), soya bean meal (35 g/L), glucose (15 g/L), peptone (2 g/L), KNO_3_ (0.5 g/L), (NH_4_)_2_SO_4_ (0.5 g/L,) NH_4_Cl (1 g/L), corn powder (15 g/L), CoCl_2_ (0.01 g/L), and CaCO_3_ (6 g/L).

### Construction of *gen*B4 disruption plasmid

The *gen*B4 gene was disrupted via pD2925-mediated double-crossover recombination. DNA isolation and manipulation were performed as described by Sambrook et al. [23]. Additional file 14: Table S1 lists the primers used in the present study. Primers were designed using the biosynthetic gene-cluster sequence for gentamicin (GenBank accession number: JQ975418.1). Primers B4up 1 and B4up 2 were used to amplify a 1434-bp fragment containing the upstream sequence of *gen*B4. Primers B4dn 1 and B4dn 2 were used to amplify a 1666-bp fragment containing the downstream sequence and the last 375 bp of *gen*B4. The two fragments were cloned separately into pMD 18-T (Takara, Japan) and were then excised from the resulting plasmids using *Hin*dIII-*Xba*I and *Xba*I-*Kpn*I. The excised products containing the upstream and downstream fragments of *gen*B4 were then ligated with the *Hin*dIII-*Kpn*I fragment of pD2925 to yield pD2925B4 for an in-frame deletion of *gen*B4 (Fig. 2).

### Construction of *gen*B4 disruption strain

The disruption plasmid, pD2925B4, was introduced into *E*. *coli* ET12567/pUZ8002 via the CaCl_2_ method and then into wild-type *M. echinospora* via conjugational transfer [24]. After incubation at 37 °C for 24 h, each dish was overlaid with 1 mL of sterile water containing apramycin at a final concentration of 20 μg/mL. Since pD2925B4 contains an apramycin-resistance gene, the exconjugants were selected as follows: the apramycin-resistant (for the first crossover event) phenotype was first selected, and the apramycin-sensitive (the second crossover event) phenotype was then selected to isolate the *gen*B4-disruption strain. The exconjugants were subsequently incubated at 37 °C for 7 days to select for homologous recombinants (for the first crossover event) and were identified by PCR using the primers B4Y1, B4Y2, B4Y3, and B4Y4. Then, the exconjugants were cultured on antibiotic-free medium for sporulation, and the cycle was repeated three times to enhance the probability of recombination. Single clones were replica-plated onto the apramycin-containing plates, as well as on plates without antibiotic for sporulation. The apramycin-sensitive strains were selected based on the growth conditions of the clones on the two different plates (the second crossover event), and the expected disruption genotype was identified by PCR using the primers, B4Y1 and B4Y4. The selected strain was named *M. echinospora* Δ*gen*B4. We used the same method to generate *M. echinospora* Δ*gen*KΔ*gen*B4.

### Complementation of disruption mutants

The pEAP1 plasmids were used to construct gene-complementation vectors. The *gen*B4 was amplified from chromosomal DNA from the original strain using the primers, B4up and B4dn. Additionally, the PCR product was inserted into the *Nco*I and *Xho*I sites of pPT2925. The cloned gene contained the *Phrd*B promoter, and the *To* terminator was digested with XbaI and SpeI and inserted into pEAP1 to generate pEAP1B4. The complementation plasmids were verified by sequencing and were then introduced individually into *M. echinospora* Δ*gen*B4 and *M. echinospora* Δ*gen*KΔ*gen*B4 by conjugation. The plasmids were integrated into *M. echinospora* chromosomal DNA via site-specific recombination. Complemented exconjugants were identified by erythromycin resistance (100 μg/mL) and were confirmed by PCR (Fig. 6).

### Antibiotic isolation and analysis

The pH of the culture broth was adjusted to 2.0 with H_2_SO_4_. The acidified broth was agitated for 30 min and was then centrifuged (14,500×*g*; 10 min). The pH of the supernatant was readjusted to 6.8 with NH_4_OH. This pre-treated supernatant was then centrifuged (14,500×*g*; 10 min) again. The supernatant was applied onto strongly acidic resin 001 × 7 (Amicogen (China) Biopharm Co., Ltd), and bound substances were eluted with 2 mol/L NH_4_OH. A second cation-exchange chromatography step was performed on the weakly acidic resin, D152 (Amicogen [China] Biopharm Co., Ltd). Bound substances were removed by gradient elution with NH_4_OH (from 0.1 to 1.0 mol/L).

Elutant from the strongly acidic resin 001 × 7 was used as the sample for reversed-phase high-performance liquid chromatography (RP-HPLC) with ELSD. The sample was determined based on HPLC-ELSD, using a reverse-phase C18 column with an evaporation temperature of 45 °C and nitrogen pressure of 3.5 bar, with a mobile phase of 0.2 mol/L trifluoroacetic acid–methanol (92:8) at a 0.6 mL/min flow rate. Authentic standard gentamicin and sisomicin were purchased from NICPBP (National Institute for the Control of Pharmaceutical and Biological Products).

### Cloning of *gen*B4 gene for expression in *E. coli*

Gene *gen*B4 was amplified from the genomic DNA of *M. echinospora* by PCR (primers B4up-A and B4dn). The PCR products were digested with NdeI and XhoI, purified by gel extraction (Vazyme), and inserted into plasmid pET28a (+) to generate pET28aB4. The resulting constructs were verified by DNA sequencing.

### Overexpression and purification of recombinant proteins

*Escherichia coli* BL21(DE3) cells containing the recombinant plasmids were cultured in LB broth containing kanamycin (25 μg/mL) at 37 °C to absorption at 600 nm of 0.5 to 0.8 and expression induced by IPTG (0.1 mM) at 16 °C with shaking overnight. Cells were harvested and resuspended in binding buffer (0.5 M NaCl, 5 mM imidazole, 20 mM Tris–HCl [pH 8.0]). The recombinant protein was released by sonication for 10 min using a 3 s on/5 s off cycle. Clarified cell lysate was passed through a column of Ni^2+^-charged His-Bind resin (GE Healthcare). After washing the column with washing buffer (0.5 M NaCl, 10-50 mM imidazole, 20 mM Tris–HCl [pH 8.0]). N-His6-tagged recombinant proteins were eluted with elution buffer (0.5 M NaCl, 250 mM imidazole, 20 mM Tris–HCl [pH 8.0]). Imidazole in the eluted protein solutions was removed by buffer exchange using Amicon Ultracentrifugal filters (Millipore). The purified proteins were stored at − 20 °C. The identities of the purified proteins were confirmed by SDS-PAGE, UV–Vis absorbance analysis. Protein concentrations were determined using Bradford protein dye reagent.

### Enzyme assays

Assay mixtures for GenB4 (500 μL) contained substrate (2 mM), purified GenB4 (15 μM), KPi buffer (50 mM, pH 8.0, KOH), and several other associated chemicals if it needed, like exogenous PLP (1 mM), amino donor (2 mM). Incubations were at 30 °C for 2–4 h and quenched by addition of equal volume chloroform followed by centrifugation to remove protein. The supernatants were analyzed by HPLC-ELSD. For NaBH_4_ or NaBD_4_ mediated reductions, NaBH_4_ or NaBD_4_ was added excessively into the GenB4 catalyzed assay solutions (already quenched) and reacted on ice for 45 min.

## Supplementary information

**Additional file 1: Figure S1.** In-frame deletion of genB4 in M. echinospora. (a) Schematic representation of the in-frame deletions in wild-type *M. echinospora*; (b) Confirmation of *M. echinospora* △*gen*B4 by PCR with the primers, B4Y1 and B4Y4. The arrows indicate the expected size of the PCR fragments in the wild type and mutants. (c) Confirmation of *M. echinospora* △*gen*K△*gen*B4 by PCR with the primers, B4Y1 and B4Y4. The arrows indicate the expected size of the PCR fragments in the wild type and mutants.

**Additional file 2: Figure S2.** (**1**) and (**6**) reduced with NaBH_4_. (**1**) and (**6**) had the same retention times in HPLC-ELSD when separated by cation-exchange chromatography. (**1**) was reduced by NaBH_4_, whereas (**6**) was not.

**Additional file 3: Figure S3.** Mass-spectra analysis of the intermediates in the mutants.

**Additional file 4: Figure S4.** Mass-spectra analysis of the intermediates in the mutants.

**Additional file 5: Figure S5.**^1^H NMR and ^13^C NMR spectroscopic analyses of intermediate (**1**). (a) ^1^H NMR spectroscopic analysis of intermediate (**1**). ^1^H NMR (600 MHz, D_2_O) δ 6.30 (t, *J* = 4.1 Hz, 1H), 5.49 (s, 1H), 4.96 (d, *J* = 3.7 Hz, 1H), 4.10 (dd, *J* = 10.9, 3.6 Hz, 1H), 3.94–3.89 (m, 1H), 3.86 (d, *J* = 12.7 Hz, 2H), 3.69–3.61 (m, 2H), 3.46–3.39 (m, 2H), 3.39–3.32 (m, 2H), 2.79 (d, *J* = 5.3 Hz, 3H), 2.59 (s, 2H), 2.49 (dt, *J* = 20.1, 4.2 Hz, 1H), 2.43 (dt, *J* = 12.6, 4.2 Hz, 1H), 2.26 (s, 3H), 1.82 (q, *J* = 12.7 Hz, 1H), 1.22 (s, 3H). (b) ^13^C NMR (151 MHz, D_2_O) δ 195.94 (s), 146.75 (s), 113.70 (s), 101.19 (s), 97.10 (s), 83.14 (s), 80.08 (s), 73.35 (s), 69.83 (s), 67.62 (s), 66.24 (s), 63.36 (s), 48.15 (s), 45.69 (s), 38.65 (s), 34.38 (s), 27.52 (s), 24.45 (s), 23.99 (s), 20.80 (s).

**Additional file 6: Figure S6.** Complementation of the *gen*B4 disrupting strain. (a) Map of the genetic complementation vector, pEAP1B4. The phrdb1 and B4dn are primers used to check the complementation strains. (b) Confirmation of the complementation strain by PCR. The arrows indicate the expected size of the PCR fragments in the original strain and the mutants.

**Additional file 7: Figure S7.** HPLC-ELSD analysis of fermentation production by complementation strains and the original strains.

**Additional file 8: Figure S8.** Characterization of purified recombinant GenB4. (a) SDS-PAGE analysis of purified GenB4 (51.9 kDa). The production of N-His6-tagged GenB4 was carried out in *E. coli* BL21(DE3). The acrylamide percentage of the SDS-PAGE gels was 12%. (b) UV–vis absorption spectrum of the purified recombinant proteins, His6-GenB4.

**Additional file 9: Figure S9.**^1^H NMR and ^13^C NMR spectroscopic analyses of compound (**8**) reduced with NaBH_4_ or NaBD_4_. (a) ^1^H NMR spectroscopic analysis of compound (**8**) reduced with NaBD_4_. ^1^H NMR (600 MHz, D_2_O) δ 5.40 (d, *J* = 3.5 Hz, 1H), 5.01 (d, *J* = 3.7 Hz, 1H), 4.14 (dd, *J* = 10.9, 3.7 Hz, 1H), 3.95–3.92 (m, 1H), 3.91 (d, *J* = 12.9 Hz, 1H), 3.81–3.76 (m, 1H), 3.72 – 3.66 (m, 3H), 3.56 (d, *J* = 1.3 Hz, 1H), 3.56 – 3.53 (m, 1H), 3.48 (ddd, *J* = 11.9, 6.2, 3.3 Hz, 4H), 3.44–3.37 (m, 2H), 2.83 (s, 3H), 2.45 (dt, *J* = 12.6, 4.2 Hz, 1H), 1.96–1.92 (m, 1H), 1.88 (dd, *J* = 12.6, 4.1 Hz, 1H), 1.84 (t, *J* = 8.7 Hz, 1H), 1.72 (dd, *J* = 14.0, 2.8 Hz, 1H), 1.48–1.41 (m, 1H), 1.26 (s, 3H). (b) ^13^C NMR spectroscopy analysis of the compound (**8**) reduced with NaBD_4_. ^13^C NMR (151 MHz, D_2_O) δ 101.14, 96.49, 83.43, 80.38, 73.63, 72.02, 71.10, 69.83, 64.76 (d, *J* = 432.03 Hz), 63.32, 62.44, 49.50, 49.06, 48.81, 34.42, 27.80, 23.78, 21.23, 20.82.

**Additional file 10: Figure S10.** Mass-spectra analysis of the transamination product of 6′-deamino-6′-oxogentamincin C1a (**8**).

**Additional file 11: Figure S11.** Analysis of the effect of amino donors on transamination of 6′-deamino-6′-oxogentamincin C1a (**8**) to gentamicin C1a (**9**). L-Tyr, L-Lys, L-Gln, L-Met, and L-Glu were separately added in the GenB4-catalyzed reactions with exogenous PLP.

**Additional file 12: Figure S12.** Analysis of the effect of different aminotransferases on transamination of 6′-deamino-6′-oxogentamincin C1a (**8**) to gentamicin C1a (**9**). GenB1, GenB2, GenB3, and GenB4 were separately added after 1 h of incubation of the GenB4 reaction system containing exogenous PLP and the amino donor, L-Glu.

**Additional file 13: Figure S13.** Mass-spectra analysis of (**11**) and (**10**).

**Additional file 14: Table S1.** Primers used in the present study.

## Data Availability

The datasets used and/or analyzed during the current study are available from the corresponding author on reasonable request.

## References

[1] Mathews A, Bailie GR (1987). Clinical pharmacokinetics, toxicity and cost effectiveness analysis of aminoglycosides and aminoglycoside dosing services. J Clin Pharm Ther.

[2] Fourmy D, Recht MI, Blanchard SC (1996). Structure of the a site of *Escherichia coli* 16S ribosomal RNA complexed with an aminoglycoside antibiotic. Science.

[3] Shaw KJ, Rather PN, Hare RS (1993). Molecular genetics of aminoglycoside resistance genes and familial relationships of the aminoglycoside-modifying enzymes. Microbiol Rev.

[4] Ramirez MS, Tolmasky ME (2010). Aminoglycoside modifying enzymes. Drug Resist Updates.

[5] Wagman GH, Oden EM, Weinstein MJ (1968). Differential chromatographic bioassay for the gentamicin complex. Appl Microbiol.

[6] Guo J, Huang F, Huang C, Duan X, Jian X, Leeper F (2014). Specificity and promiscuity at the branch point in gentamicin biosynthesis. Chem Biol.

[7] Kim HJ, Mccarty RM, Ogasawara Y (2013). GenK-catalyzed C-6′ methylation in the biosynthesis of gentamicin: isolation and characterization of a cobalamin-dependent radical SAM enzyme. J Am Chem Soc.

[8] Li S, Guo J, Reva A (2018). Methyltransferases of gentamicin biosynthesis. Proc Natl Acad Sci.

[9] Shao L, Junsheng C, Chunxia W (2013). Characterization of a key aminoglycoside phosphotransferase in gentamicin biosynthesis. Bioorg Med Chem Lett.

[10] Lu Y, Dong X, Liu S (2009). Characterization and identification of a novel marine *Streptomyces* sp. produced antibacterial substance. Mar Biotechnol.

[11] Marquez JA, Wagman GH, Testa RT (1976). A new broad spectrum aminoglycoside antibiotic, G-52, produced by *Micromonospora zionensis*. J Antibiot.

[12] Daniels PJL, Jaret RS, Nagabhushan TL (1976). The structure of antibiotic G-52, a new aminocyclitol-aminoglycoside antibiotic produced by *Micromonospora zionensis*. J Antibiot.

[13] Kase H, Shimura G, Iida T (1982). Biotransformation of sisomicin and verdamicin by *Micromonospora sagamiensis*. Agric Biol Chem.

[14] Yuan YZ, Zhang M, Fan XL (2013). Analysis of impurities in vertilmicin sulfate by liquid chromatography ion-trap mass spectrometry. J Pharm Biomed Anal.

[15] Li B, Schepdael AV, Hoogmartens J (2011). Mass spectrometric characterization of gentamicin components separated by the new European Pharmacopoeia method. J Pharm Biomed Anal.

[16] Yuan YZ, Zhao X, Zhang M (2013). Impurity profiling of micronomicin sulfate injection by liquid chromatography–ion trap mass spectrometry. J Pharm Biomed Anal.

[17] Hanessian S, Szychowski J, Maianti JP (2009). Synthesis and comparative antibacterial activity of verdamicin C2 and C2a. A new oxidation of primary allylic azides in dihydro[2H]pyrans. Org Lett.

[18] Sucipto H, Kudo F, Eguchi T (2012). The last step of kanamycin biosynthesis: unique deamination reaction catalyzed by the α-ketoglutarate-dependent nonheme iron dioxygenase KanJ and the NADPH-dependent reductase KanK. Angew Chem Int Ed Engl.

[19] Wang M, Zhao Q, Zhang Q (2017). Differences in PLP-dependent cysteinyl processing lead to diverse S-functionalization of lincosamide antibiotics. Sci Found China.

[20] Ban YH, Song MC, Hwang JY, Shin HL, Kim HJ, Hong SK (2019). Complete reconstitution of the diverse pathways of gentamicin B biosynthesis. Nat Chem Biol.

[21] Gu Y, Ni X, Ren J (2015). Biosynthesis of epimers C2 and C2a in the gentamicin c complex. ChemBioChem.

[22] Kim JY, Suh JW, Kang SH (2008). Gene inactivation study of gntE reveals its role in the first step of pseudotrisaccharide modifications in gentamicin biosynthesis. Biochem Biophys Res Commun.

[23] Russell DW, Sambrook J (2001). Molecular cloning: a laboratory manual.

[24] Kieser T, Bibb M, Buttner M, Chater K, Hopwood D (2000). Practical streptomyces genetics.

[25] Macneil DJ, Gewain KM, Ruby CL (1992). Analysis of *Streptomyces avermitilis* genes required for avermectin biosynthesis utilizing a novel integration vector. Gene.

[26] Janssen GR, Bibb MJ (1993). Derivatives of pUC18 that have BglII sites flanking a modified multiple cloning site and that retain the ability to identify recombinant clones by visual screening of *Escherichia coli* colonies. Gene.

