# Supplementary figures and images for: The bifunctional enzyme, GenB4, catalyzes the last step of gentamicin 3′,4′-di-deoxygenation via reduction and transamination activities
Source: Microb Cell Fact. 2020 Mar 10;19:62. doi: 10.1186/s12934-020-01317-0 (PMC7063804; doi:10.1186/s12934-020-01317-0)

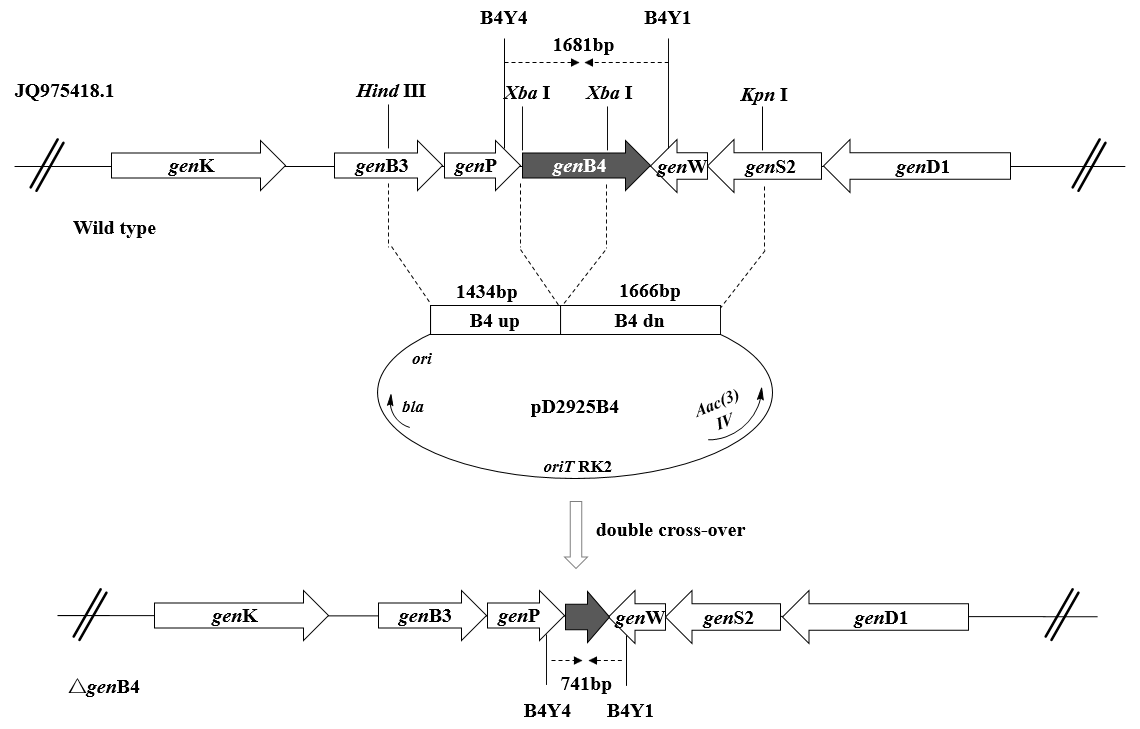

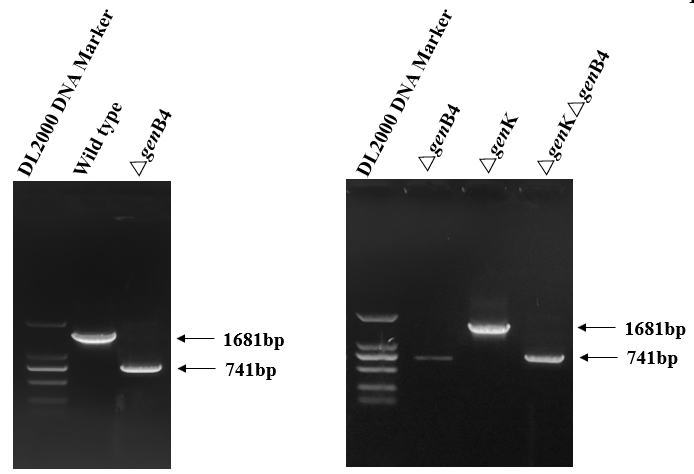


b

c

a

Supplement: Supplementary file 1 — Additional file 1: Figure S1. In-frame deletion of genB4 in M. echinospora. (a) Schematic representation of the in-frame deletions in wild-type M. echinospora; (b) Confirmation of M. echinospora △genB4 by PCR with the primers, B4Y1 and B4Y4. The arrows indicate the expected size of the PCR fragments in the wild type and mutants. (c) Confirmation of M. echinospora △genK△genB4 by PCR with the primers, B4Y1 and B4Y4. The arrows indicate the expected size of the PCR fragments in the wild type and mutants. [file 12934_2020_1317_MOESM1_ESM.docx]

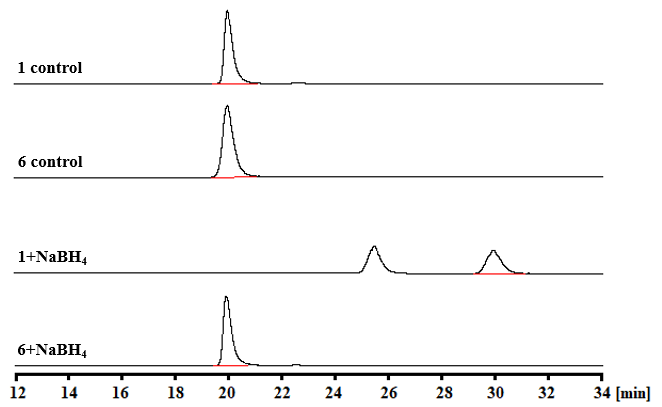

Supplement: Supplementary file 2 — Additional file 2: Figure S2. (1) and (6) reduced with NaBH4. (1) and (6) had the same retention times in HPLC-ELSD when separated by cation-exchange chromatography. (1) was reduced by NaBH4, whereas (6) was not. [file 12934_2020_1317_MOESM2_ESM.docx]

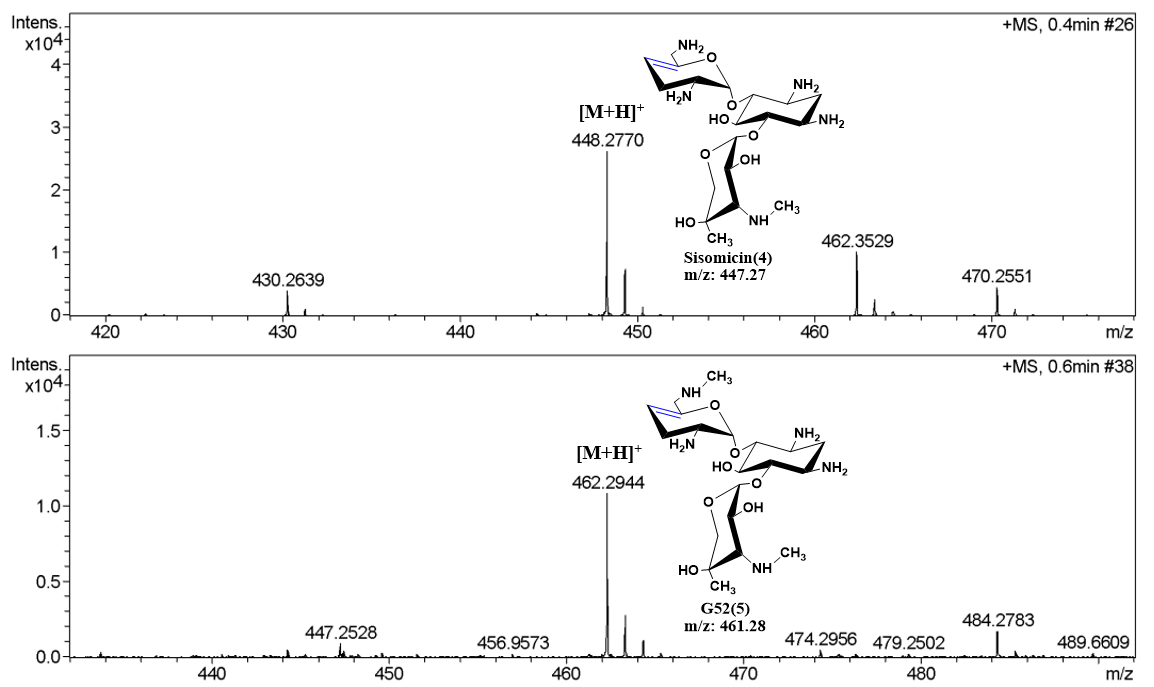

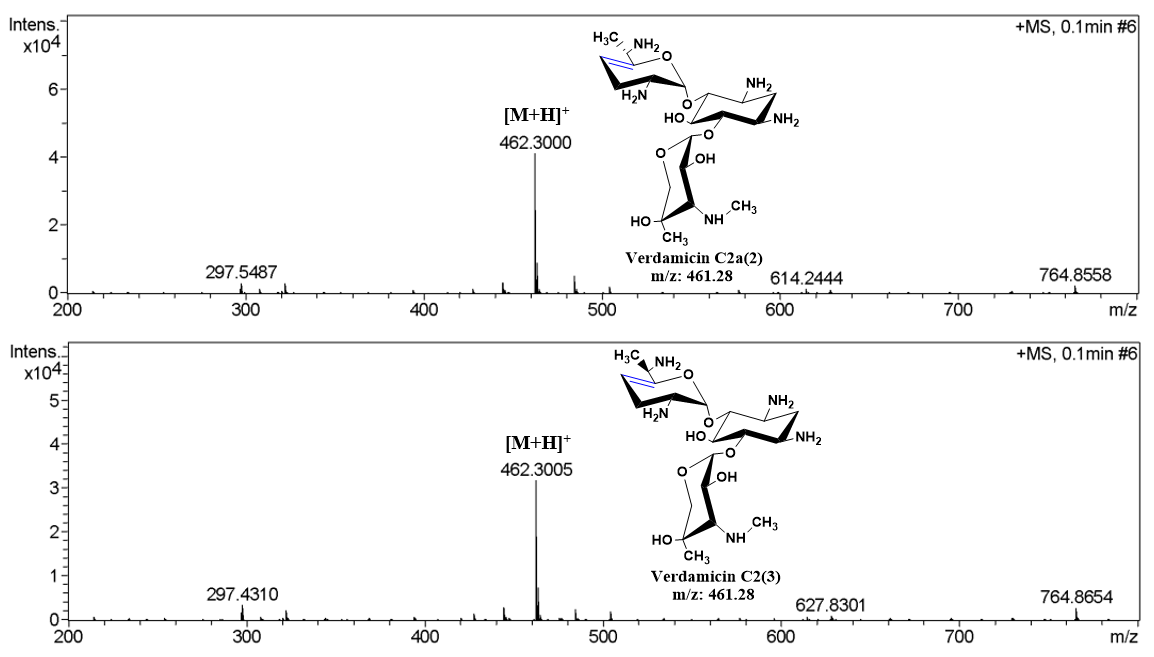


d

c

b

a

Supplement: Supplementary file 3 — Additional file 3: Figure S3. Mass-spectra analysis of the intermediates in the mutants. [file 12934_2020_1317_MOESM3_ESM.docx]

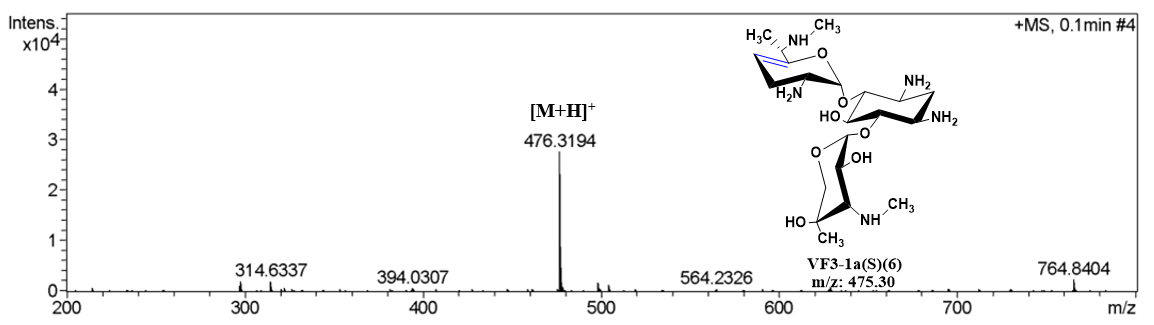

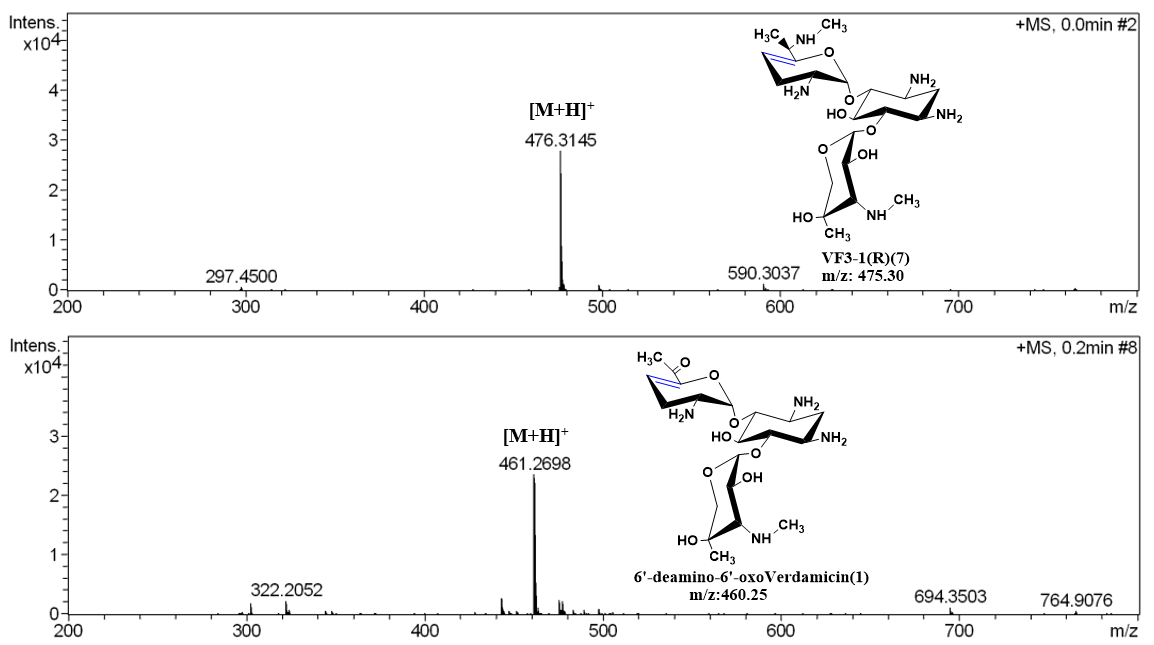


c

b

a

Supplement: Supplementary file 4 — Additional file 4: Figure S4. Mass-spectra analysis of the intermediates in the mutants. [file 12934_2020_1317_MOESM4_ESM.docx]

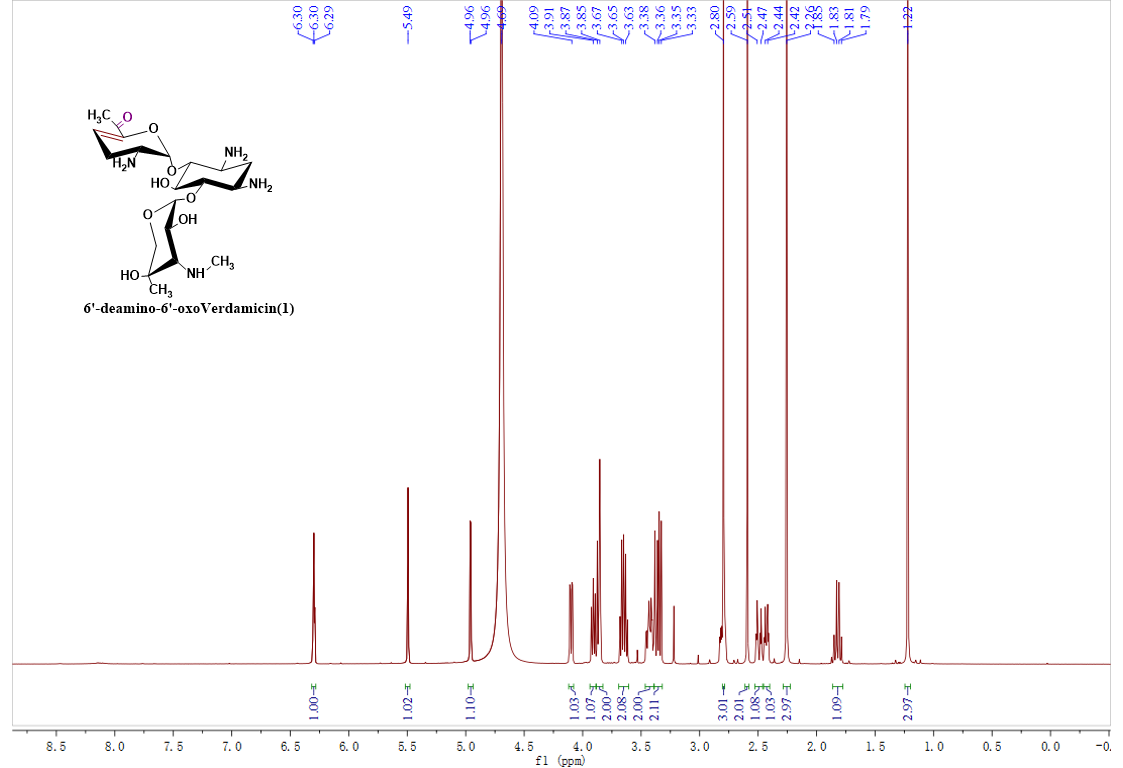

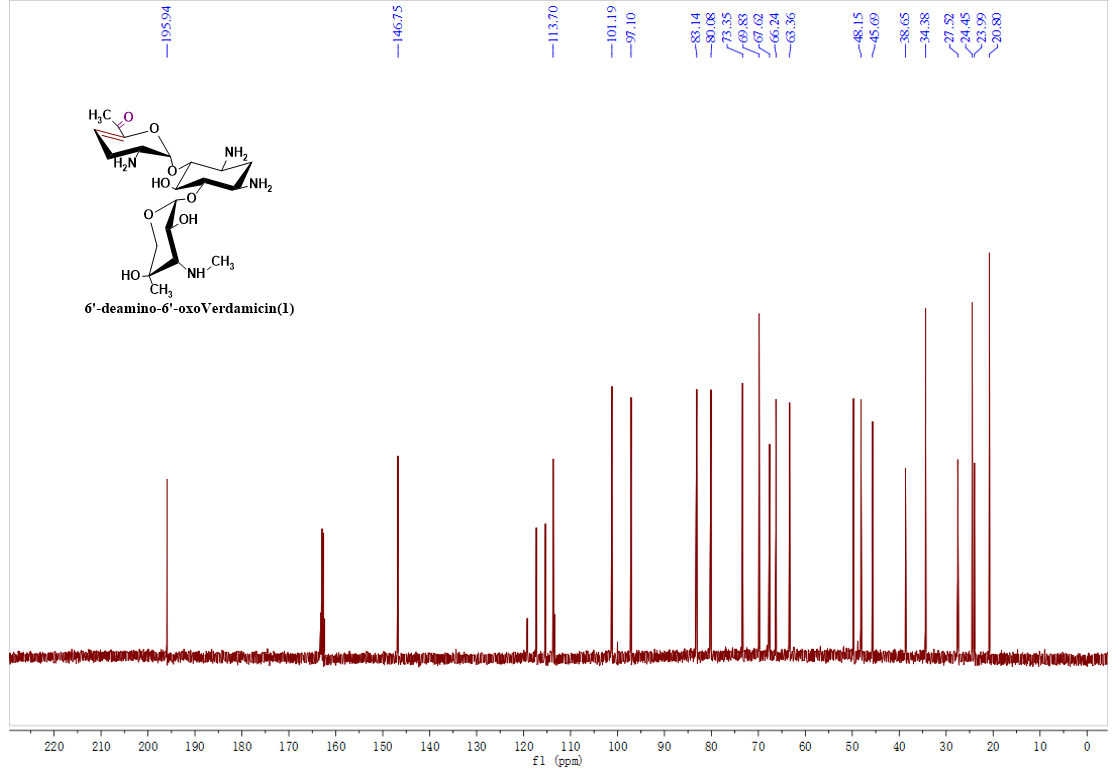


b

a

Supplement: Supplementary file 5 — Additional file 5: Figure S5.1H NMR and 13C NMR spectroscopic analyses of intermediate (1). (a) 1H NMR spectroscopic analysis of intermediate (1). 1H NMR (600 MHz, D2O) δ 6.30 (t, J = 4.1 Hz, 1H), 5.49 (s, 1H), 4.96 (d, J = 3.7 Hz, 1H), 4.10 (dd, J = 10.9, 3.6 Hz, 1H), 3.94–3.89 (m, 1H), 3.86 (d, J = 12.7 Hz, 2H), 3.69–3.61 (m, 2H), 3.46–3.39 (m, 2H), 3.39–3.32 (m, 2H), 2.79 (d, J = 5.3 Hz, 3H), 2.59 (s, 2H), 2.49 (dt, J = 20.1, 4.2 Hz, 1H), 2.43 (dt, J = 12.6, 4.2 Hz, 1H), 2.26 (s, 3H), 1.82 (q, J = 12.7 Hz, 1H), 1.22 (s, 3H). (b) 13C NMR (151 MHz, D2O) δ 195.94 (s), 146.75 (s), 113.70 (s), 101.19 (s), 97.10 (s), 83.14 (s), 80.08 (s), 73.35 (s), 69.83 (s), 67.62 (s), 66.24 (s), 63.36 (s), 48.15 (s), 45.69 (s), 38.65 (s), 34.38 (s), 27.52 (s), 24.45 (s), 23.99 (s), 20.80 (s). [file 12934_2020_1317_MOESM5_ESM.docx]

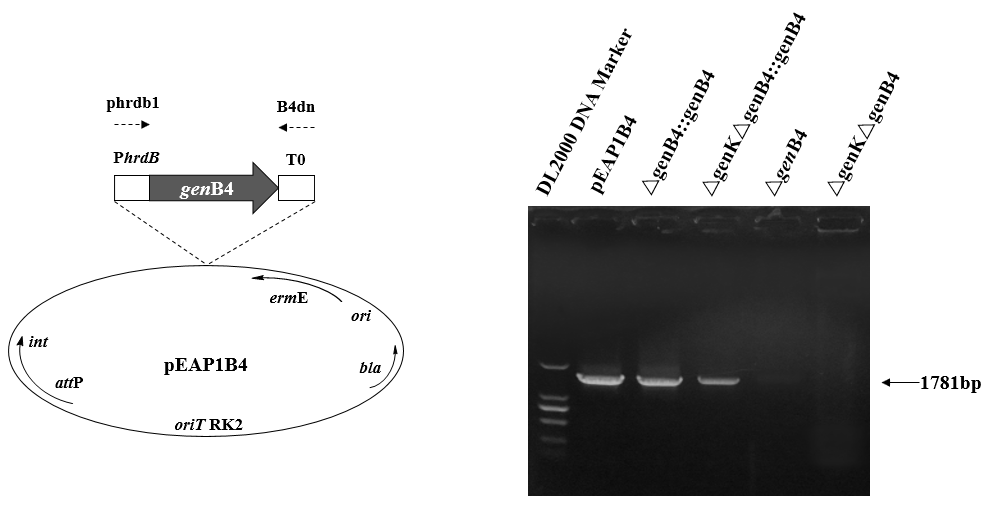


b

a

Supplement: Supplementary file 6 — Additional file 6: Figure S6. Complementation of the genB4 disrupting strain. (a) Map of the genetic complementation vector, pEAP1B4. The phrdb1 and B4dn are primers used to check the complementation strains. (b) Confirmation of the complementation strain by PCR. The arrows indicate the expected size of the PCR fragments in the original strain and the mutants. [file 12934_2020_1317_MOESM6_ESM.docx]

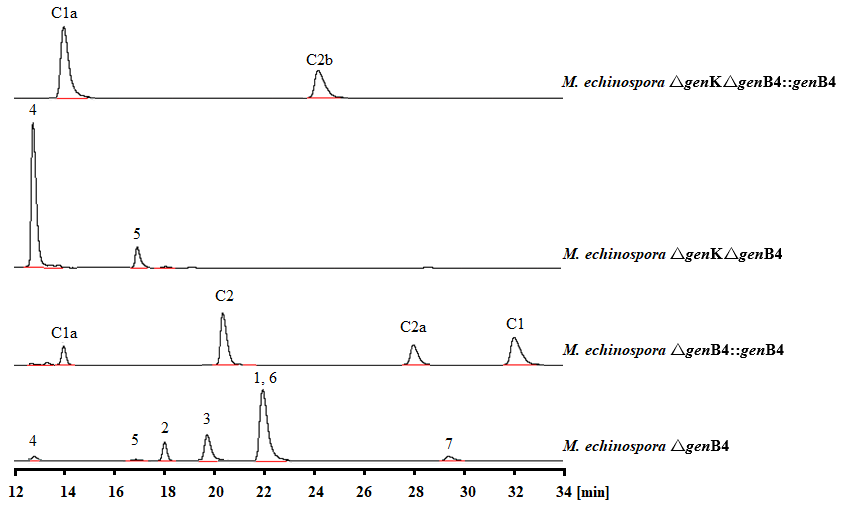

Supplement: Supplementary file 7 — Additional file 7: Figure S7. HPLC-ELSD analysis of fermentation production by complementation strains and the original strains. [file 12934_2020_1317_MOESM7_ESM.docx]

b

a


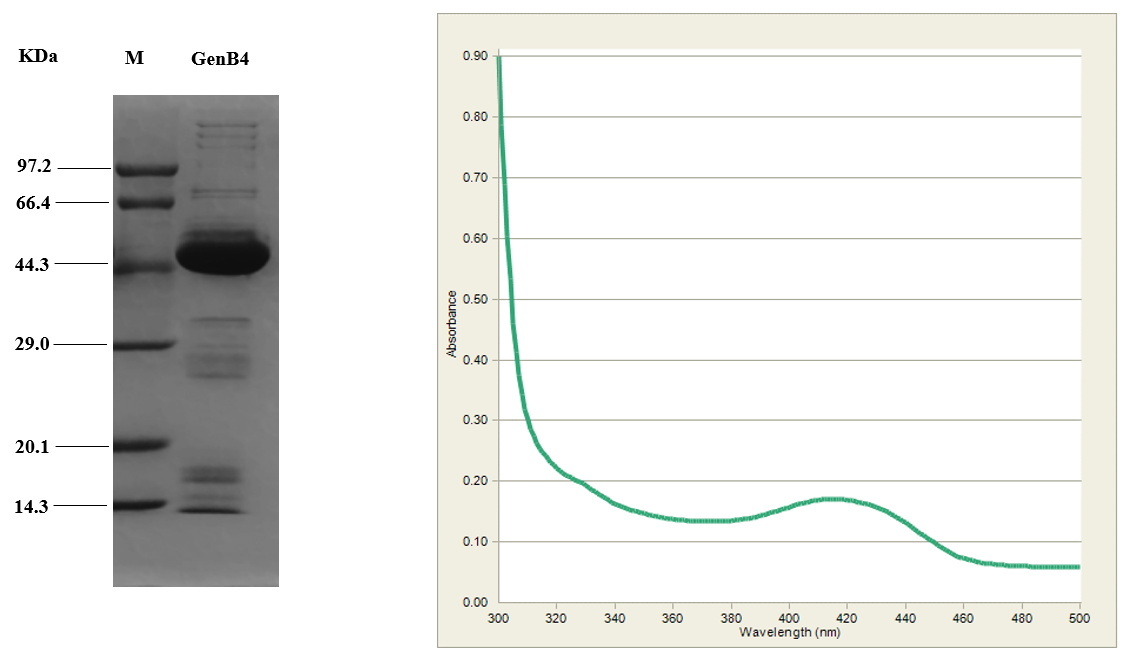

Supplement: Supplementary file 8 — Additional file 8: Figure S8. Characterization of purified recombinant GenB4. (a) SDS-PAGE analysis of purified GenB4 (51.9 kDa). The production of N-His6-tagged GenB4 was carried out in E. coli BL21(DE3). The acrylamide percentage of the SDS-PAGE gels was 12%. (b) UV–vis absorption spectrum of the purified recombinant proteins, His6-GenB4. [file 12934_2020_1317_MOESM8_ESM.docx]

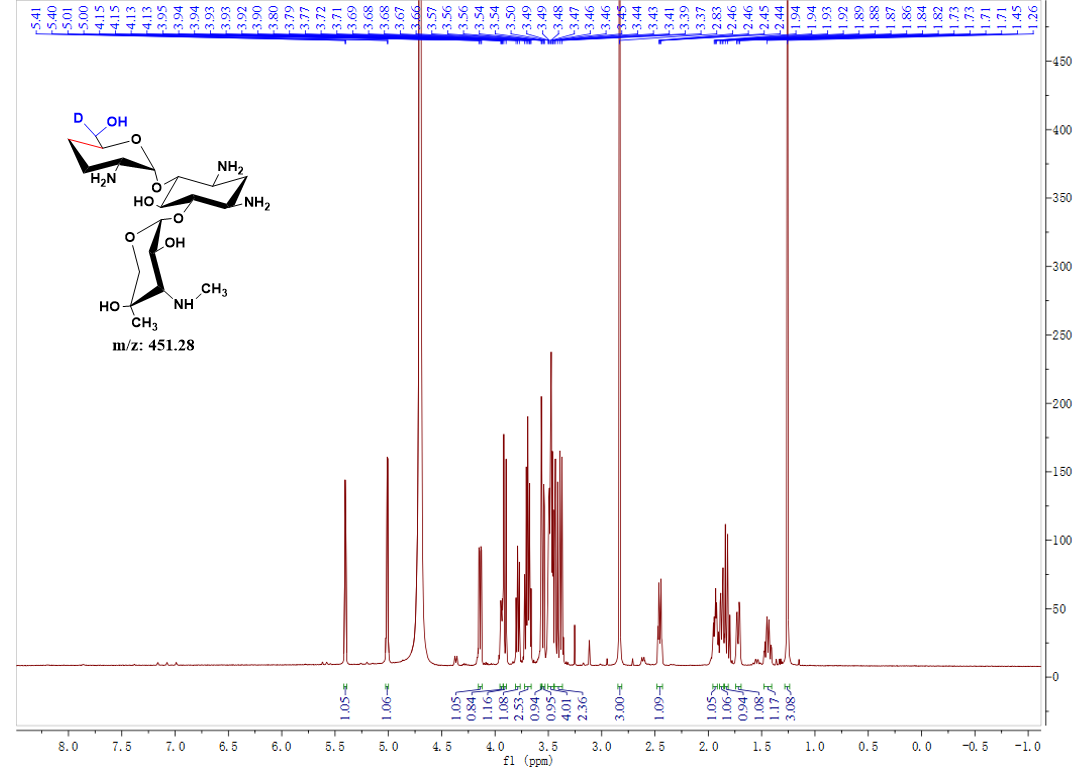

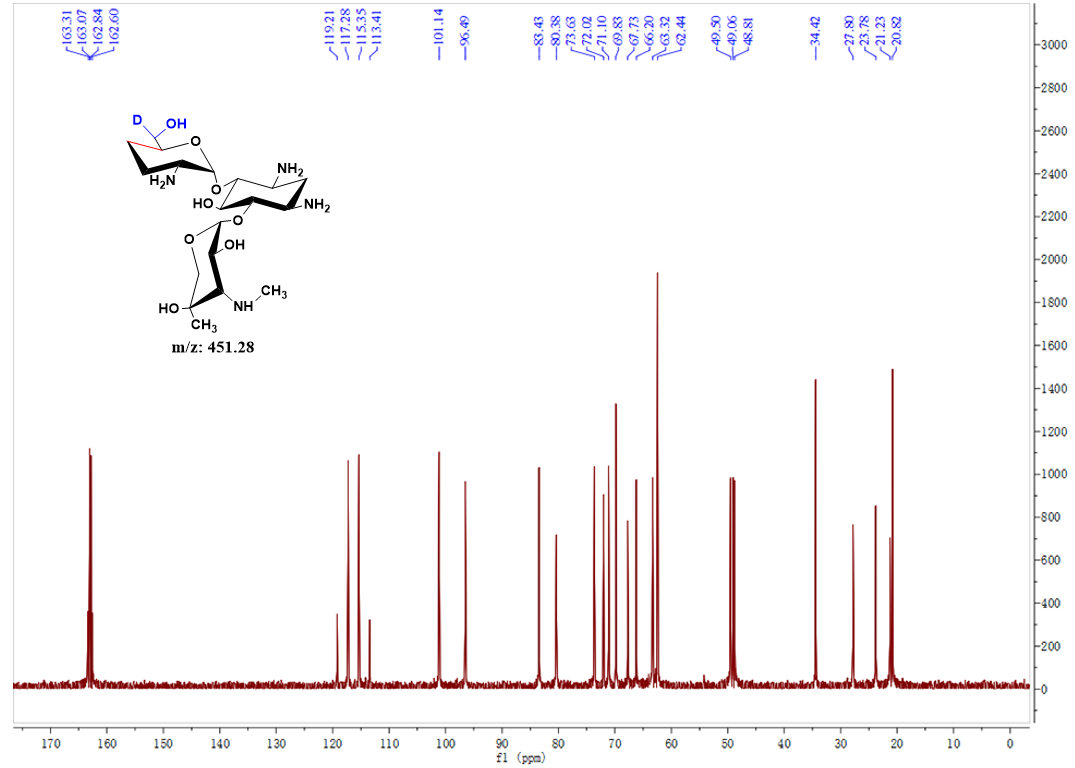


b

a

Supplement: Supplementary file 9 — Additional file 9: Figure S9.1H NMR and 13C NMR spectroscopic analyses of compound (8) reduced with NaBH4 or NaBD4. (a) 1H NMR spectroscopic analysis of compound (8) reduced with NaBD4. 1H NMR (600 MHz, D2O) δ 5.40 (d, J = 3.5 Hz, 1H), 5.01 (d, J = 3.7 Hz, 1H), 4.14 (dd, J = 10.9, 3.7 Hz, 1H), 3.95–3.92 (m, 1H), 3.91 (d, J = 12.9 Hz, 1H), 3.81–3.76 (m, 1H), 3.72 – 3.66 (m, 3H), 3.56 (d, J = 1.3 Hz, 1H), 3.56 – 3.53 (m, 1H), 3.48 (ddd, J = 11.9, 6.2, 3.3 Hz, 4H), 3.44–3.37 (m, 2H), 2.83 (s, 3H), 2.45 (dt, J = 12.6, 4.2 Hz, 1H), 1.96–1.92 (m, 1H), 1.88 (dd, J = 12.6, 4.1 Hz, 1H), 1.84 (t, J = 8.7 Hz, 1H), 1.72 (dd, J = 14.0, 2.8 Hz, 1H), 1.48–1.41 (m, 1H), 1.26 (s, 3H). (b) 13C NMR spectroscopy analysis of the compound (8) reduced with NaBD4. 13C NMR (151 MHz, D2O) δ 101.14, 96.49, 83.43, 80.38, 73.63, 72.02, 71.10, 69.83, 64.76 (d, J = 432.03 Hz), 63.32, 62.44, 49.50, 49.06, 48.81, 34.42, 27.80, 23.78, 21.23, 20.82. [file 12934_2020_1317_MOESM9_ESM.docx]

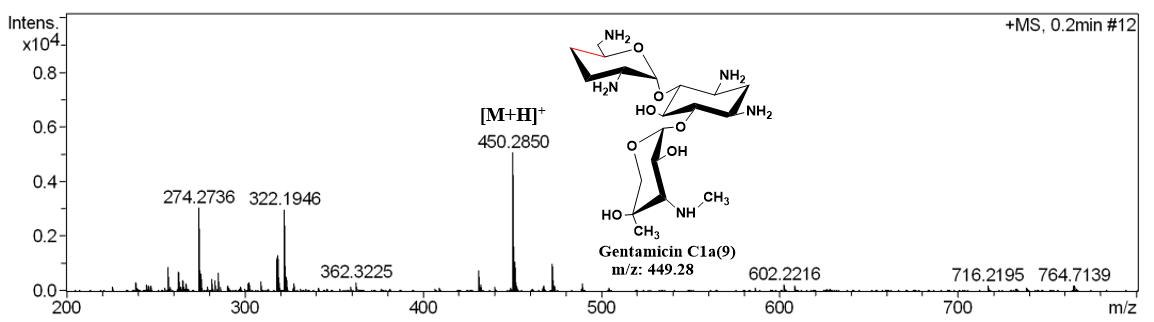

Supplement: Supplementary file 10 — Additional file 10: Figure S10. Mass-spectra analysis of the transamination product of 6′-deamino-6′-oxogentamincin C1a (8). [file 12934_2020_1317_MOESM10_ESM.docx]

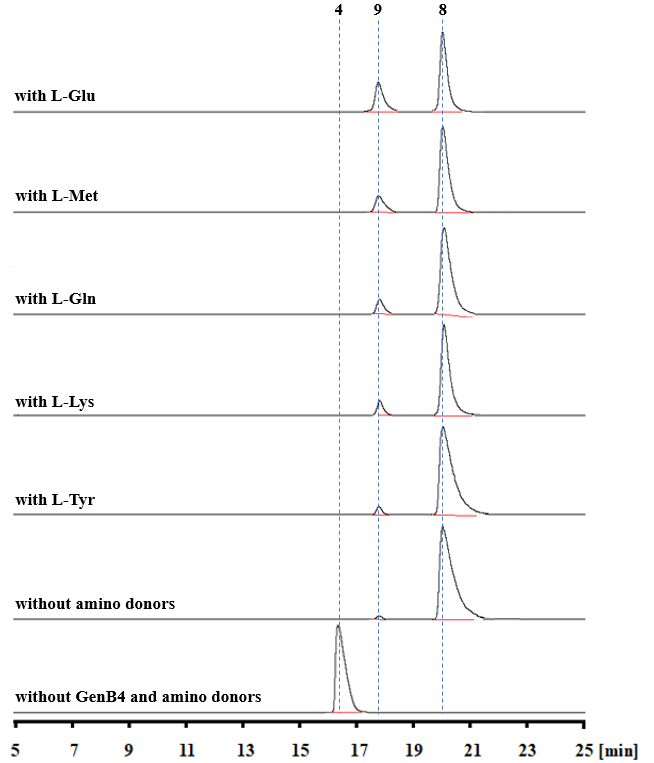

Supplement: Supplementary file 11 — Additional file 11: Figure S11. Analysis of the effect of amino donors on transamination of 6′-deamino-6′-oxogentamincin C1a (8) to gentamicin C1a (9). L-Tyr, L-Lys, L-Gln, L-Met, and L-Glu were separately added in the GenB4-catalyzed reactions with exogenous PLP. [file 12934_2020_1317_MOESM11_ESM.docx]

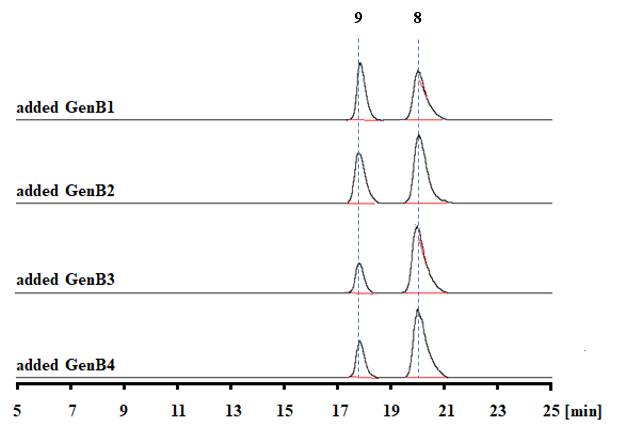

Supplement: Supplementary file 12 — Additional file 12: Figure S12. Analysis of the effect of different aminotransferases on transamination of 6′-deamino-6′-oxogentamincin C1a (8) to gentamicin C1a (9). GenB1, GenB2, GenB3, and GenB4 were separately added after 1 h of incubation of the GenB4 reaction system containing exogenous PLP and the amino donor, L-Glu. [file 12934_2020_1317_MOESM12_ESM.docx]

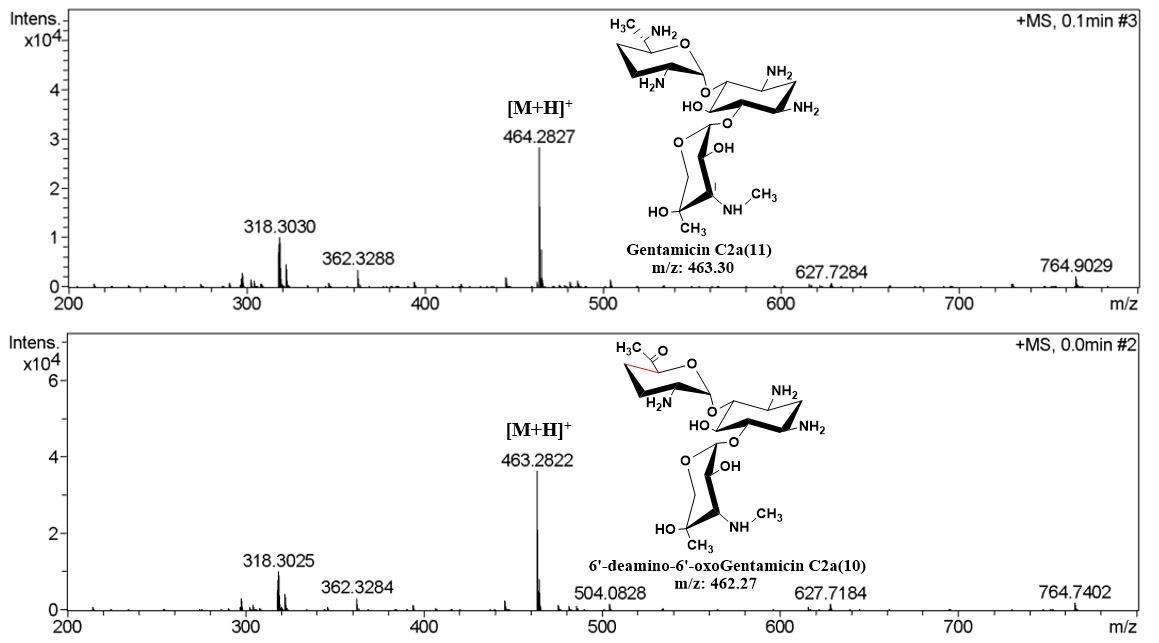


**b**

**a**

Supplement: Supplementary file 13 — Additional file 13: Figure S13. Mass-spectra analysis of (11) and (10). [file 12934_2020_1317_MOESM13_ESM.docx]
