# Supplementary material for: The bifunctional enzyme, GenB4, catalyzes the last step of gentamicin 3′,4′-di-deoxygenation via reduction and transamination activities
Source: Microb Cell Fact. 2020 Mar 10;19:62. doi: 10.1186/s12934-020-01317-0 (PMC7063804; doi:10.1186/s12934-020-01317-0)
Supplement: Supplementary file 14 — Additional file 14: Table S1. Primers used in the present study. [file 12934_2020_1317_MOESM14_ESM.docx]

| Primer | Oligonucleotide sequences (5' to 3') | | Restriction site |
| --- | --- | --- | --- |
| B4up 1 | | AAGCTTGTTATGGATGTGCTCGACACG | *Hind*Ⅲ |
| B4up 2 | | TCTAGACTCGATCAACTCACGGTAGTTCAT | *Xba*I |
| B4dn1 | | TCTAGAGCGTTGATGTTCGACGTGGTG | *Xba*I |
| B4dn2 | | GGTACCGGCAACCAGGTCGCCGACATG | *Kpn*I |
| B4Y1 | | CGAGCACCTGGCGACGAAGATGCG | - |
| B4Y2 | | GGCTGACGTGGTGGTGGTCAGCAAGG | - |
| B4Y3 | | GCCGGCATCGTGCACGTCAAC | - |
| B4Y4 | | GCGTCAACCAGCACAAGCTGACC | - |
| B4up | | CCATGGACTACCGTGAGTTGATCGAG | *Nco*Ⅰ |
| B4up-A | | CATATGAACTACCGTGAGTTGATCGAG | *Nde*Ⅰ |
| B4dn | | CTCGAGGGATCAGTTCTGTGCGGGAAC | *Xho*Ⅰ |
| phrdb1 | | CTCTAGACCGCCTTCCGCCGGAACG | *Xba*I |
| phrdb2 | | CCATGGACAACCTCTCGGAACGTTG | *Nco*I |
